# Real-World Estimation of First- and Second-Line Treatments for Diffuse Large B-Cell Lymphoma Using Health Insurance Data: A Belgian Population-Based Study

**DOI:** 10.3389/fonc.2022.824704

**Published:** 2022-02-28

**Authors:** Willem Daneels, Michael Rosskamp, Gilles Macq, Estabraq Ismael Saadoon, Anke De Geyndt, Fritz Offner, Hélène A. Poirel

**Affiliations:** ^1^ Department of Hematology, Ghent University Hospital, Ghent, Belgium; ^2^ Faculty of Medicine and Health Sciences, Ghent University, Ghent, Belgium; ^3^ Cancer Research Institute, Ghent University, Ghent, Belgium; ^4^ Belgian Cancer Registry, Brussels, Belgium

**Keywords:** DLBCL - diffuse large B cell lymphoma, population-based cancer registry, health insurance database, first- and second-line therapy, R-CHOP, hematopoietic stem cell transplantation, comorbidities, real-world studies (RWS)

## Abstract

We determined first- and second-line regimens, including hematopoietic stem cell transplantations, in all diffuse large B cell lymphoma (DLBCL) patients aged ≥20 yr (n = 1,888), registered at the Belgian Cancer Registry (2013–2015). Treatments were inferred from reimbursed drugs, and procedures registered in national health insurance databases. This real-world population-based study allows to assess patients usually excluded from clinical trials such as those with comorbidities, other malignancies (12%), and advanced age (28% are ≥80 yr old). Our data show that the majority of older patients are still started on first-line regimens with curative intent and a substantial proportion of them benefit from this approach. First-line treatments included full R-CHOP (44%), “incomplete” (R-)CHOP (18%), other anthracycline (14%), non-anthracycline (9%), only radiotherapy (3%), and no chemo-/radiotherapy (13%), with significant variation between age groups. The 5-year overall survival (OS) of all patients was 56% with a clear influence of age (78% [20–59 yr] versus 16% [≥85 yr]) and of the type of first-line treatments: full R-CHOP (72%), other anthracycline (58%), “incomplete” (R-)CHOP (47%), non-anthracycline (30%), only radiotherapy (30%), and no chemo-/radiotherapy (9%). Second-line therapy, presumed for refractory (7%) or relapsed disease (9%), was initiated in 252 patients (16%) and was predominantly (71%) platinum-based. The 5-year OS after second-line treatment without autologous stem cell transplantation (ASCT) was generally poor (11% in ≥70 yr versus 17% in <70 yr). An ASCT was performed in 5% of treated patients (n = 82). The 5-year OS after first- or second-line ASCT was similar (69% versus 66%). After adjustment, multivariable OS analyses indicated a significant hazard ratio (HR) for, among others, age (HR 1.81 to 5.95 for increasing age), performance status (PS) (HR 4.56 for PS >1 within 3 months from incidence), subsequent malignancies (HR 2.50), prior malignancies (HR 1.34), respiratory and diabetic comorbidity (HR 1.41 and 1.24), gender (HR 1.25 for males), and first-line treatment with full R-CHOP (HR 0.41) or other anthracycline-containing regimens (HR 0.72). Despite inherent limitations, patterns of care in DLBCL could be determined using an innovative approach based on Belgian health insurance data.

## 1 Introduction

Diffuse large B-cell lymphoma (DLBCL) is the most common mature B-cell lymphoma, making up about 25%–30% of all lymphoma subtypes in developed countries. For the Belgian population, a median age at diagnosis of 71 years with a crude incidence of 7.8 and age-standardized incidence rate using the European (2013) standard population (ESR2013) of 7.5/100.000 per year was reported in 2018 and 2019, respectively (1–4). Risk stratifications have been developed such as the International Prognostic Index (IPI) and several adaptations (R-IPI, age adjusted-IPI, National Comprehensive Cancer Network (NCCN)-IPI) incorporating tumor stage, lactate dehydrogenase (LDH) level, extranodal involvement, WHO performance status (PS), and age (1, 5, 6).

By gene expression profiling, the cell of origin (COO) can be distinguished as being of germinal center (germinal center B-cell, GCB), activated B-cell subtype (ABC), or non-classifiable. The ABC subtype is generally associated with a worse prognosis (1, 7). However, in routine practice, the cell of origin (COO) is usually determined by immunohistochemistry (IHC) as a proxy (GCB versus non-GCB) due to the unavailability of gene expression profiling. Unfortunately, this approach comes with several disadvantages such as a lower specificity (8). Cytogenetics allow for the identification of “high-grade B-cell lymphoma (HGBCL), with rearrangements of *MYC* and *BCL2* and/or *BCL6*,” which is a subgroup with a worse prognosis (8). Overexpression of BCL2 has been identified as a negative prognostic marker (9). In about 30% of cases, both MYC and BCL2 are overexpressed without concomitant translocations in so-called double-expressing lymphomas (DEL), another high-risk group (10). More recently, several genetically defined subtypes of DLBCL have been proposed, based on the combination of various molecular aberrations, which might lead to more individualized treatments upon validation (11–13).

The current standard of care for DLBCL is still immuno-chemotherapy with R-CHOP (rituximab [R], cyclophosphamide, hydroxydaunorubicin, vincristine, prednisolone) followed by involved field radiotherapy (IFRT) in certain risk groups. A remission can be achieved in about 80% of patients, which is durable in 70% of cases, resulting in a 5-year overall survival (OS) of 65% in the R-CHOP era (1, 14). Attempts to improve on R-CHOP by adding novel agents have mostly been disappointing (14–16). Patients who experience primary refractory or relapsed disease have a poor prognosis with limited therapeutic options at that point (1, 17). Whenever possible, these patients should be included in clinical trials. Outside of clinical trials, fit patients are generally offered salvage regimens containing rituximab and platinum derivatives, followed by high-dose chemotherapy (HDC), and autologous stem cell transplantation (ASCT). Unfit patients will be offered either similar/less toxic salvage regimens without ASCT or alternatively palliative regimens. Some patients relapsing after ASCT can currently be offered CAR-T cell therapy (chimeric antigen receptor T cells), allogeneic hematopoietic stem cell transplantation (AlloSCT), or novel therapies such as tafasitamab, polatuzumab vedotin, or selinexor (6, 18–20).

However, a significant proportion of patients are unfit for these predominantly intensive treatments because of advanced age and/or comorbidities (21). In real life, the majority of DLBCL patients are older than 65 years of age at diagnosis and a significant proportion have a prior history of other malignancies and/or other comorbidities (2, 22–29). These groups are usually excluded from clinical trials resulting in uncertainty about their optimal clinical management. This underscores the growing interest for real-world population-based studies, to compare the results of randomized clinical trials, with a more representative and unselected population.

With this study, we describe the real-world pattern of care in adult (≥20 yr) DLBCL patients, diagnosed in Belgium between 2013 and 2015, with a specific focus on patients aged ≥60 yr, using the Belgian Cancer Registry (BCR) and health insurance databases, to infer treatment modalities as well as comorbidities.

## 2 Materials and Methods

### 2.1 The Belgian Cancer Registry and Accessible Databases

The Belgian Cancer Registry (BCR) collects, processes, and analyzes data on all new cancers diagnosed in Belgian residents, by independent collection of double input: oncological care programs and pathology reports. Near-complete coverage is presumed due to combined reporting in nearly 90% of DLBCL cases (4). The BCR is authorized by law to use the National Social Security Identification Number, making it possible to link these data to national administrative health insurance data from the Intermutualistic Agency (IMA). The IMA centralizes details on all healthcare reimbursements of all Belgian citizens (30). Vital status was available until April 2021 through linkage with the national Crossroads Bank for Social Security, providing a follow-up of 5–8 years for all patients. All health records were pseudonymized prior to analysis.

### 2.2 In- and Exclusion Criteria

Using the diagnostic code 9680/3 from the third edition of the International Classification of Diseases for Oncology (ICD-O-3) (32), we included all new diagnoses of adult (≥20 yr) DLBCL (including B-cell lymphoma unclassifiable), with features intermediate between DLBCL and Burkitt lymphoma (31) and high-grade B-cell lymphoma (HGBCL) [NOS/with *MYC* and *BCL2* and/or *BCL6* rearrangements) (14)] in Belgium between January 1, 2013, and December 31, 2015 (n = 2,139). The final cohort included 1,888 patients after step-wise exclusion of 251 cases due to no available survival data (n = 17), non-Belgian residents (n = 2), no IMA records (n = 38), suspicion of posttransplant lymphoproliferative disorder after prior solid organ/stem cell transplantation (PTLD; n = 33), primary central nervous system lymphoma (PCNSL; n = 158), acute lymphoblastic leukemia (ALL; n = 1), mantle cell lymphoma (MCL; n = 0), or primary mediastinal B-cell lymphoma (PMBCL; n = 2).

### 2.3 Extraction of Biomarkers

Besides structured files from pathology laboratories, the BCR also receives free-text pathology reports. The latter were used to extract the status of ten main biomarkers (obtained by manual annotations and verified by natural language processing (NLP) automatic extraction). These included expression levels of immunohistochemistry (IHC) markers (CD10, BCL6, IRF4, BCL2, BCL6, MYC, KI-67), cell of origin (COO) classification as determined by the Hans algorithm (33), and gene rearrangements (*MYC*, *BCL2*, *BCL6*) by fluorescence *in situ* hybridization (FISH). Expression of IHC markers was defined positive or negative as described in the pathology report or, when available, using cutoff values for the individual IHC markers according to international guidelines (e.g., ≥40% MYC-positive nuclei and ≥50% for BCL2 expression) (10, 14).

### 2.4 Extraction of Clinical Data

The ECOG/WHO performance status (PS) and Ann Arbor stage were retrieved from the records of oncological care programs and were available in 85% and 66% of cases, respectively. Information regarding B-symptoms and extra-nodular involvement was only poorly available and not considered for analysis.

### 2.5 Extraction of Data on Comorbidities

Because the modified Charlson Comorbidity Index (CCI) could not be calculated for 2015, respiratory, cardiovascular, and diabetic comorbidities were assessed for each patient using health insurance data of reimbursed drugs as previously published (34). The BCR gathers information on all new cancer diagnoses in Belgium; hence, we could identify patients having multiple malignancies. Patients were considered to have another tumor if a diagnosis of another malignancy (excluding non-melanoma skin cancer), with an incidence date within 5 years prior to DLBCL diagnosis or thereafter, was registered at the BCR. Additionally, patients without another cancer diagnosis but who received other non-lymphoma-specific chemotherapy within the study period were also identified and considered for outcome analyses.

### 2.6 Identification of Treatment Regimens

Health insurance data provided a timestamped list of all reimbursed drugs and (medical) procedures per patient. We considered all drugs and procedures within the timeframe of 30 days prior to and 2 years after diagnosis. This window was determined based on the assumption that some drugs might be administered before a definitive diagnosis was made, potential small deviations between the billing and administration date, and that most relapses in DLBCL occur within 2 years (35–38).

For chemotherapy, we included all drugs with the ATC code ‘L01’ (“Antineoplastic and immunomodulating agents” from the Anatomical Therapeutic Chemical (ATC) Classification System) (39). These drugs are further classified according to specific Belgian CNK codes (Code Nationa(a)l Kode), which allowed us to identify the specific brand, dose, and distribution form (40). An in-house algorithm was set up to define the treatment regimens based on the timed combination of different drugs and administration route. For example, registration of rituximab, cyclophosphamide, and vincristine within a 12-day period was considered as 1 cycle of R-CVP. The addition of doxorubicin within the same timeframe would be considered as R-CHOP. The number of cycles and cycle duration was based on the interval between these drug administrations. Modifications to the initial regimen during treatment could be identified, and the first-line regimen was reclassified based on the predominant regimen.

By selecting for nomenclature codes (a coded list of all medical performances that are entitled for (partial) reimbursement by the mandatory national health insurance), we identified autologous and allogeneic hematopoietic stem cell transplantations (HSCTs) and all forms of external beam radiotherapy (RT). Data on transplantations were available until December 2019 and have been cross-validated and completed with data from the Belgian Transplant Registry (BTR), which is hosted by the BCR. Data on HSCT performed for presumed acute myeloid leukemia (AML), acute lymphoblastic leukemia (ALL), or myelodysplastic syndrome (MDS) were excluded from our analyses.

We defined refractory and relapsed disease as initiation of any second-line regimen within or beyond 12 weeks from the end of the last first-line treatment administration, respectively. Consolidation regimens, such as in the LNH03-2B protocol (16), and central nervous system (CNS) prophylaxis [e.g., high-dose methotrexate (HD MTX)] within 6 weeks after the end of first-line treatment were still considered to be part of the first-line regimen. Intrathecal (IT) and intravenous (IV) administration of MTX could be distinguished based on the CNK codes.

During our study period, the standard-of-care regimen recommended by ESMO/NCCN/BHS (6, 18, 35) for all DLBCL patients was R-CHOP for 6–8 cycles but based on more recent findings from the FLYER (41), SWOG S0014 (42), and LNH09-1B (43) trials, excellent results can be achieved in patients with low-risk limited stage disease with only 4 cycles (or even 3 with IFRT). We therefore considered full R-CHOP as ≥6 cycles (n = 793) or ≥4 for Ann Arbor stage I (n = 33). For statistical analyses, treatments were hierarchically grouped into 6 main categories according to their most important components: full R-CHOP (≥6 or ≥4 cycles if Ann Arbor = I, including R-miniCHOP); incomplete (R-)CHOP (<6 cycles or <4 if Ann Arbor = I, and CHOP without R); other anthracycline-containing regimens (e.g., (R-)ACVBP, (R-)CHOP-like, intensified regimens); non-anthracycline-containing regimens (e.g., R-CVP, bendamustine-containing regimens, palliative treatments); only radiotherapy; and no chemo/radiotherapy. Second-line treatments were regrouped into 4 main categories: platinum-containing; non-platinum-containing; bendamustine-containing; and palliative regimens. An in-depth manual revision of more than 400 cases was performed to fine-tune the algorithm.

### 2.7 Statistical Analyses

Analyses were performed using the SAS 9.4 software package (SAS institute, Cary, NC). Uni- and multivariable survival analyses were based on Cox models. For the multivariable model, we have included all our variables of interest without interaction between them. To avoid a problem of collinearity, we decided to include PS and not Ann Arbor stage in the final multivariable model, as the former had proportionally fewer missing values. For Ann Arbor stage, PS, center volume, BCL2 overexpression on IHC, and COO, we have considered an interaction with a timepoint binary variable (equal to 0 before the considered timepoint and 1 after it) because the proportional hazard assumption (44) was not fulfilled for the whole study period. Consequently, for these variables, hazard ratios were estimated for two distinct periods following the incidence. Because treatments (and likewise the diagnosis of subsequent tumors) occurred after the DLBCL incidence date, the starting point of our study, the different treatments (and subsequent tumors) were considered as time-dependent variables to avoid an immortal time bias (45). The hazard ratio of each treatment compares the group of patients who received the treatment with all other patients (including patients with other treatments). Tests for statistical significance were 2-sided at an alpha = 0.05 level of significance and 95% confidence intervals [95% CI]. Relative survival is calculated as the ratio of the observed survival in a group of patients to the expected survival (obtained with Ederer II method) (4) in a comparable group of individuals from the general Belgian population matched on age, sex, region, and calendar period.

## 3 Results

### 3.1 Population Characteristics

We analyzed 1,888 newly diagnosed DLBCL patients with a male/female ratio of 1.2. The median age was 72 years (interquartile range 61–80 yr [IQR]) with 28% of patients aged 80 years or older. Patient characteristics and prognostics markers by age category are detailed in **Supplementary Table 1**. Information on PS was missing in 15% of cases but, when available, was generally deemed good (0 or 1) in 82% of all, and in 72% of patients ≥85 yr. In 12% of cases, another malignancy was registered at the BCR (**Supplementary Figure 1** shows the exact distribution and timing with regard to the DLBCL diagnosis). In 20 patients, 2 or more malignancies (excluding the DLBCL) were registered within the considered timeframe. We did not find an increased standardized incidence ratio (SIR) of prior malignancies compared to the general population when stratified by gender, region, 5-year age category, and incidence year. Respiratory, diabetic, and cardiovascular comorbidities increased with age. Ann Arbor stage was distributed similarly across all age groups, when corrected for the increased number of missing data with advancing age. Information on the cell of origin (COO) was available in 63% of cases with an even distribution of GCB and non-GCB subtypes (32 and 31%). The distribution of COO was similar across all age categories, Ann Arbor stages, PS, comorbidities, and first-line treatments. BCL2 was overexpressed in 79% of evaluable cases. Of only 16% evaluable cases, 49% were double-expressor lymphomas (DEL). Information on *MYC*, *BCL2*, and *BCL6* rearrangements was available in only 11%, 11%, and 8% of cases, respectively. These limited cases demonstrated 20% of isolated *MYC* rearrangements, and 8.7% of “HGBCL, with rearrangements of *MYC* and *BCL2* and/or *BCL6*” according to the latest WHO classification (14).

### 3.2 Overall Survival Stratified by Age Groups

**Figure 1:**
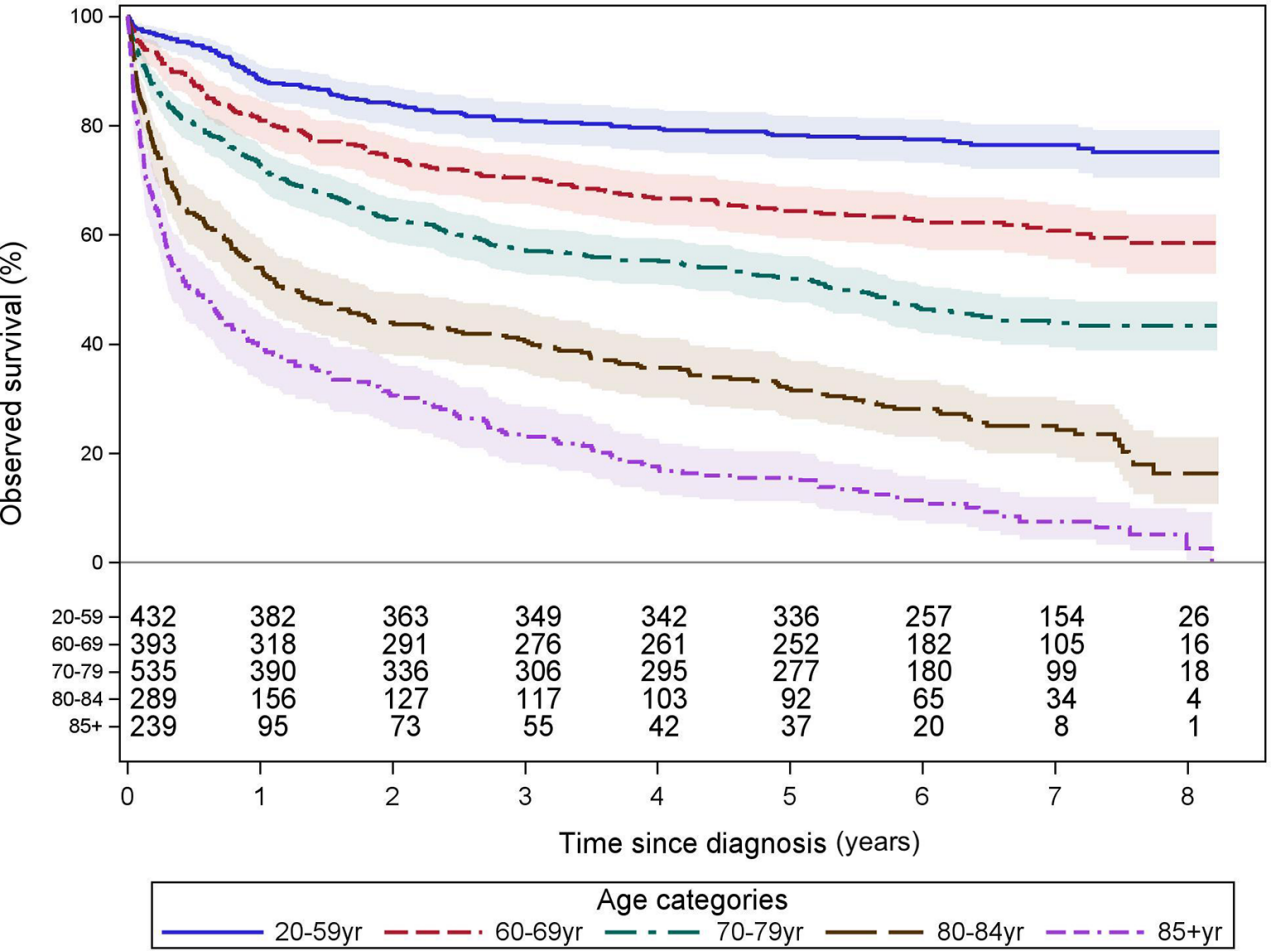
Observed survival by age categories. These Kaplan–Meier curves show the observed survival from time of diagnosis, of all 1,888 patients, grouped into 5 clinically relevant age categories associated with a significantly different overall survival from time of diagnosis. The numbers of patients at risk are tabled below the curves. Colored areas represent the 95% confidence intervals.

The 5-year OS of all patients was 56% with a clear influence of age (from 78% [20–59 yr] to 16% [≥85 yr]). Survival curves for the age categories below 55 years closely overlap. Beyond 55 years of age, survival probability decreases with age as demonstrated in **Supplementary Figure 2**. In contrast to the International Prognostic Index (IPI), which uses 60 yr as the only age cutoff, survival changed more markedly after the age of 70. We have regrouped our cohort into 5 clinically relevant categories which are of adequate size for statistical comparisons and demonstrate a different overall survival. These groups are [20–59 yr; n = 432], [60–69 yr; n = 393], [70–79 yr; n = 535], [80–84 yr; n = 289], and [≥85 yr; n = 239]. The 5-year OS (%[95% CI]) was 78 [74.0–81.8], 64 [59.4–68.9], 52 [47.6–56.1], 32 [26.5–37.2], and 16 [11.2–20.4], respectively, and longer follow-up is shown in **Figure 1**.

To correct for competing causes of death in this predominantly older population, we determined the 2- and 5-year relative survival of the whole cohort, 69% and 63%, respectively. Similar to OS, relative survival decreased with age. Relative survival according to the major patient and treatment characteristics are shown in **Supplementary Table 2**.

### 3.3 First-Line Treatments

Systemic first-line treatment was started in 85% of cases, varying from 95% in <60 yr to only 46% in ≥85 yr. These treatments contained rituximab in most cases (96%) and were predominantly (90%) anthracycline-containing regimens (considered as curative intent), even in 51% of patients ≥85 yr. The exact frequency of all first-line regimens, including concomitant use of rituximab, is shown in **Supplementary Table 3**. Treatments were regrouped into full R-CHOP (44%), “incomplete” (R-)CHOP (18%), other anthracycline (14%), non-anthracycline (9%), only RT (3%), and no chemo/RT (13%). As detailed in **Table 1**, treatments varied between age groups: younger patients were more frequently treated with anthracycline-containing regimens other than R-CHOP (e.g., R-ACVBP), in contrast to older patients, who were more frequently treated with non-anthracycline-containing regimens (e.g., R-CVP), radiotherapy alone, and no systemic treatment at all. The median [IQR] delay from diagnosis to the start of systemic treatment or radiotherapy was 21 [13–34] days, consistent across age groups.

**Table 1:** Grouped first- and second-line treatments, including HSCT, by age group.

Age categories	N (%)	20–59 years	60–69 years	70–79 years	80–84 years	85+ years
**First-line regimens**	**N = 1,888**	**N = 432 (22.9%)**	**N = 393 (20.8%)**	**N = 535 (28.3%)**	**N = 289 (15.3%)**	**N = 239 (12.7%)**
Full R-CHOP^a^	826 (44)	210 (49)	238 (61)	261 (49)	90 (31)	27 (11)
Incomplete R-CHOP^b^	337 (18)	78 (18)	70 (18)	110 (21)	62 (21)	17 (7)
Other anthracycline^c^	271 (14)	115 (27)	45 (11)	68 (13)	30 (10)	13 (5)
Non-anthracycline^d^	162 (9)	8 (2)	16 (4)	39 (7)	46 (16)	53 (22)
Only radiotherapy^e^	47 (2)	2 (0.5)	3 (0.8)	6 (1)	12 (4)	24 (10)
No chemo/radiotherapy	245 (13)	19 (4)	21 (5)	51 (10)	49 (17)	105 (44)
**Second-line regimens**	**N = 252**	**N = 82**	**N = 71**	**N = 71**	**N = 24**	**N = 4**
Platinum-based	178 (71)	64 (78)	56 (79)	45 (63)	12 (50)	1 (25)
Cytarabine-based^f^	8 (3)	4 (5)	3 (4)	1 (1)	0 (0)	0 (0)
Anthracycline-based	17 (7)	8 (10)	2 (3)	6 (8)	1 (4)	0 (0)
Bendamustine-based	8 (3)	0 (0)	0 (0)	1 (1)	5 (21)	2 (50)
Palliative	19 (8)	1 (1)	4 (6)	11 (15)	3 (13)	0 (0)
Other^g^	22 (9)	5 (6)	6 (8)	7 (10)	3 (13)	1 (25)
% of start first line	16%	20%	19%	15%	11%	4%
% of diagnosed	13%	19%	18%	13%	8%	2%
Refractory^h^ (%first line)	111 (7)	34 (8)	35 (9)	32 (7)	8 (4)	2 (2)
Relapsed^h^ (%first line)	142 (9)	49 (12)	36 (10)	39 (8)	16 (7)	2 (2)
**HSCT**^**i**^	**N = 92**	**N = 66**	**N = 24**	**N = 2**	**N = 0**	**N = 0**
Autologous	82	56	24	2	0	0
Allogeneic	10	8	2	0	0	0

a ≥ 6 cycles (≥ 4 if Ann Arbor stage = I).

b Incomplete if < 6 cycles or < 4 if Ann Arbor stage = I or if CHOP without R.

c R-ACVBP, RA-CHOP, CHOEP, COEP, CODOX-M, HyperCVAD, CHOP-like, DHAP, DHAP-like, ICE, platinum-containing, R-MAD.

d R-monotherapy, R-CVP, bendamustine-containing, experimental and palliative regimens.

e Within 12 weeks from diagnosis, 6 additional patients received only RT > 12 weeks from diagnosis.

f Not containing platinum, anthracyclines, or bendamustine.

g Includes CNS-directed therapy, only gemcitabine-containing, experimental therapies.

h Presumed refractory of relapsed when starting the 2nd line of therapy < or >12 weeks from last administration of the first-line treatment.

i Hematopoietic stem cell transplantation, after 1st, 2nd, or further lines of therapy.

**Figure 2:**
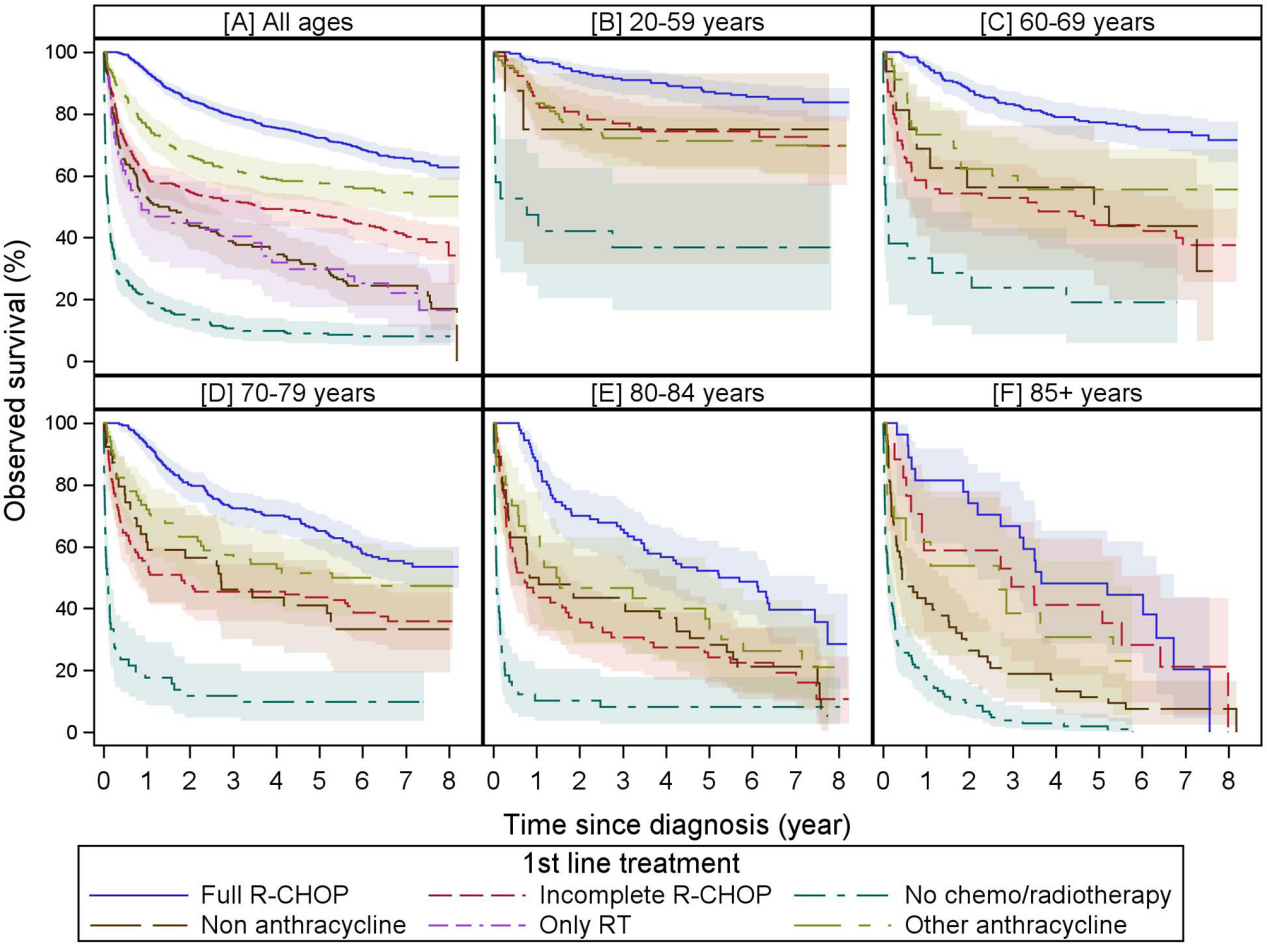
Observed survival by first-line treatment by age group. These Kaplan–Meier curves show the observed survival from diagnosis stratified by first-line treatment of all patients **(A)** and stratified by age group (20–59, 60–69, 70–79, 80–85, and 85 years+ in (**B–F**, respectively). For all age groups, full R-CHOP and “no chemo/radiotherapy” are consistently associated with the best and worst overall survivals, respectively. Immortal time bias is not taken into account as survival is presented from diagnosis and patients have to survive until the end of a treatment to be categorized as having received this treatment. While observed survival in the whole cohort for incomplete R-CHOP, other anthracycline, and non-anthracycline groups are significantly different, this is largely lost when stratified by age except in the oldest age category. Due to low numbers, overall survival for radiotherapy alone is only displayed for the whole cohort. Colored areas represent the 95% confidence intervals.

The 2- and 5-year overall survivals (%[95% CI]) vary across the first-line treatments: full R-CHOP 85 [81.9–86.8] and 72 [69.1–75.2], other anthracycline 66 [60.5–71.7] and 58 [51.8–63.5], “incomplete” R-CHOP 55 [49.4–60.0] and 47 [41.7–52.4], non-anthracycline 44 [36.1–51.3] and 30 [23.4–37.4], only radiotherapy 45 [30.2–58.1] and 30 [17.6–43.0], and no chemo/radiotherapy groups 14 [10.2–19.0] and 9.0 [5.8–13.0]. Observed survival by first-line treatment and age group is visualized in **Figure 2**.

#### 3.3.1 R-CHOP Regimens

R-CHOP was started in 1,163/1,596 (73%) of treated patients. The median [IQR] cycle interval was 21 [21–22] days, consistent across age groups. The median [IQR] number of cycles was 6 [4–8]. In 62% of R-CHOP-treated patients aged 85–89 yr, and 2 patients aged ≥90 yr, ≥6 cycles were given. However, our methodology could not discriminate R-CHOP from R-miniCHOP, the preferred regimen in patients ≥80 years old (46, 47).

In 337/1,163 cases (29%), we classified treatment as “incomplete” R-CHOP (<4 cycles (n = 178), 4–5 cycles (n = 142) if Ann Arbor stage >I, and CHOP without rituximab (n=17)).

In 40/337, a second-line regimen was started within 12 weeks, indicating primary refractory patients. Radiotherapy was applied after <4 and 4–5 R-CHOP cycles in 43/178 and 36/142 patients, respectively. Detailed patient characteristics of these different incomplete R-CHOP subgroups are shown in **Supplementary Table 4**. Importantly, when compared to the full R-CHOP cohort, patients receiving radiotherapy after incomplete R-CHOP had a similar PS (0–1 in 80%) but a higher proportion of Ann Arbor stage I–II disease (29% versus 49%).

**Figure 3:**
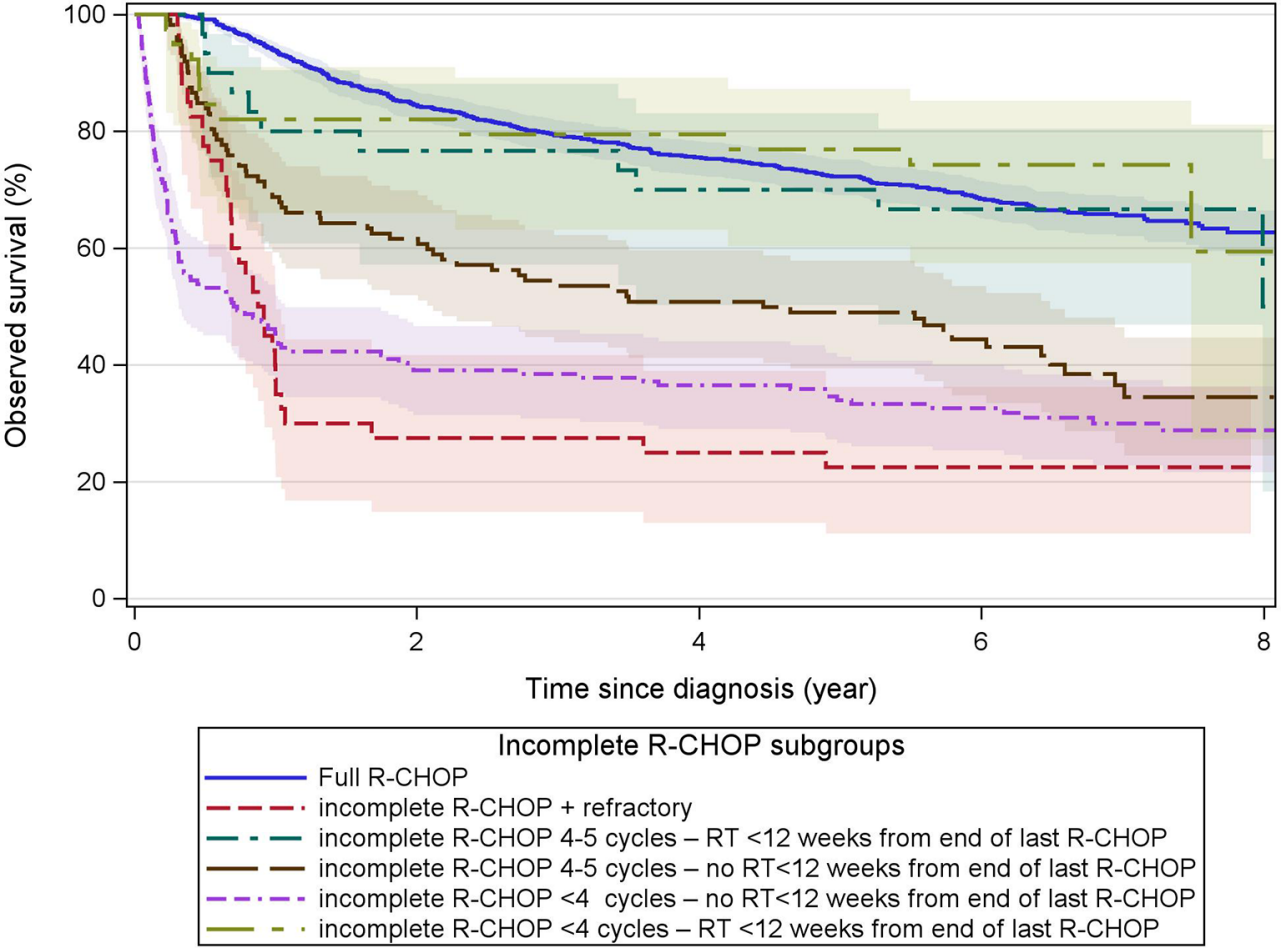
Observed survival after incomplete R-CHOP. These Kaplan–Meier curves show the observed survival from diagnosis of patients receiving first-line treatment with incomplete R-CHOP (< 6 cycles or < 4 cycles if Ann Arbor stage = I, or CHOP without R) grouped by refractory status (start of any second-line treatment within 12 weeks from the end of first-line therapy), number of R-CHOP cycles, and radiotherapy within 12 weeks from the end of the last R-CHOP cycle. Primary refractory cases had the worst survival. The overall survival of incomplete R-CHOP followed by radiotherapy (green curves) was similar to that of full-R-CHOP. Immortal time bias is not taken into account as survival is presented from diagnosis and patients have to survive until the end of a treatment to be categorized as having received this treatment. Colored areas represent the 95% confidence intervals.

The 5-year OS with incomplete R-CHOP ranged between 23% and 77% with primary refractory cases and radiotherapy groups associated with the lowest and highest OS, respectively (**Figure 3**).

#### 3.3.2 Other Anthracycline-Containing Regimens

In our cohort, 271 patients were treated in first line with anthracycline-containing regimens different from the standard R-CHOP or with platinum-based regimens (frequencies summarized in **Table 2**). Anthracycline subtypes used were doxorubicin (88%), followed by epirubicin (10%) and mitoxantrone (2%). This was a younger population compared to R-CHOP-treated patients (42% versus 25% are <60 yr). Other patient characteristics were similar and are shown in **Supplementary Table 5**. The reasons for attribution to this group are however unknown and could potentially be for DLBCL with high-risk features. The latter, combined with the younger age, could potentially explain the intermediate 5-year OS when compared to all R-CHOP-treated patients (58% versus 65%).

**Table 2:** Breakdown of other anthracycline-containing first-line regimens.

Other anthracycline-containing regimens^a^	Frequency	Percent
(R-) CHOP-like	86	32%
(R-) ACVBP	59	22%
(R-) CODOX-M/HyperCVAD	43	16%
(R-) CHOEP	43	16%
(R-) CEOP	26	10%
(R-) DHAOX	1	0.4%
(R-) DHAP	2	0.7%
(R-) ICE	1	0.4%
(R-) MAD	6	2%
Other platinum-containing regimens	4	1%

^a^This group also includes platinum-based regimens used in first-line.

#### 3.3.3 Non-Anthracycline-Containing Regimens

This group mainly consists of 93/162 patients treated with (R-)CVP, 20/162 with rituximab in monotherapy, and 23/162 with palliative regimens (**Supplementary Table 3**). Compared to full R-CHOP, this group is enriched with patients ≥80 yr (14% versus 61%) and cardiovascular comorbidities (52% versus 81%), potentially explaining, at least in part, the inferior 5-year OS of 30% (**Figure 2**).

#### 3.3.4 Radiotherapy

During our study period, 379/1,888 (20%) patients received radiotherapy of which 336/379 (89%) within 12 months from diagnosis. For 53 patients, this was the only registered treatment. We discriminated between “Early” and “Late” radiotherapy (within 12 weeks from diagnosis or thereafter). In short, 30% fell into the “early” category with 47/101 not receiving any further systemic treatment. The “early” group was enriched with older patients when compared to the “late” group, and “late” radiotherapy was performed less frequently with advancing age (26% in 20–59 yr, 20% in 60–69 yr, 30% in 70–79 yr, 17% in 80–84 yr, and 6% in ≥85 yr). When available, the Ann Arbor stage in each group was predominantly stages I–II (60%–64%) or stage IV (29–33%). The exact indications for radiotherapy are unknown but presumably include urgent decompression/pain, primary radiotherapy, or palliation in the “early” group and consolidation after first-line treatment or treatment of relapsed/refractory disease in the “late” group.

Survival of patients treated with only primary radiotherapy is poor compared to the whole cohort but nonetheless is equal to 30% at 5 years compared to only 9% for those receiving neither radiotherapy nor systemic treatment (**Figure 2**).

#### 3.3.5 No Systemic Treatment

Overall, 292 patients (15%) did not receive any lymphoma-directed systemic treatment with 53 of them receiving radiotherapy alone (see previous section). This frequency increased with age, and 65% of patients in this subgroup were ≥80 years old. Compared to the other treatment groups, information on prognostic factors like Ann Arbor stage, PS, COO, and BCL2 overexpression on IHC was more frequently missing (**Supplementary Table 5**). As expected, the survival of these patients was very poor with most patients deceased within 4 months (**Figure 2**).

#### 3.3.6 CNS-Directed Therapy

We considered any CNS-directed therapy, administered between diagnosis and 6 weeks from the end of first-line treatment, to be prophylactic. In our cohort of R-CHOP(-like)-treated patients, CNS-directed prophylaxis was administered in 19% of cases. This proportion increased with advancing Ann Arbor stage and worsening PS but decreased with advancing age (**Supplementary Table 6**). Overall survival was not significantly different. However, enrichment of younger patients in the CNS prophylaxis group is a major confounder (<70 yr in 70% versus 45%). The administration of MTX was predominantly IT (IT; n = 176; 77% versus IV; n = 55; 23%). This is in contrast to the current ESMO guidelines preferring IV MTX over IT MTX for efficacy (18). In 70/229 (31%) of cases, CNS prophylaxis was administered after completion of systemic therapy. It was impossible to determine the efficacy of CNS prophylaxis in our cohort, since we had neither information on CNS relapse nor initial CNS involvement.

### 3.4 Second-Line Treatments

A second-line therapy was initiated in 252 patients, or 16% of those starting any first-line therapy (20 to 4% decreasing with age), and was predominantly platinum-based. A summary by age, including subsequent HSCT, is shown in **Table 1**, and a more detailed analysis of the different second-line regimens by age category is shown in **Supplementary Table 7**.

**Figure 4:**
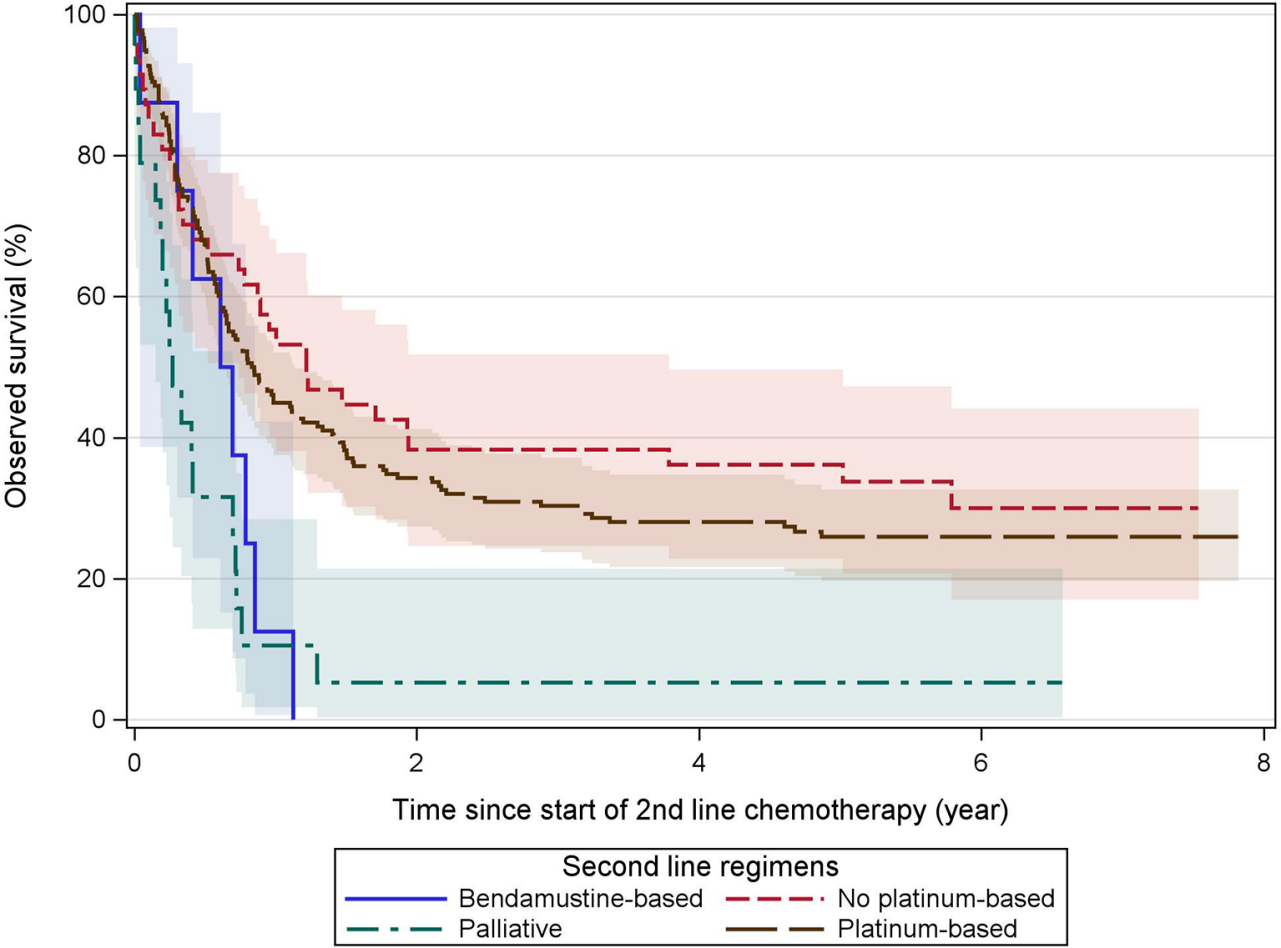
Observed survival after second-line treatment. These Kaplan–Meier curves show the observed survival from the start of second-line treatment grouped by treatment categories. Patients receiving subsequent ASCT and/or AlloSCT are included. Both platinum and non-platinum-containing regimens are associated with a similar but limited long-term overall survival. Palliative and bendamustine-containing regimens provided (nearly) no survival beyond the 1-year mark. Colored areas represent the 95% confidence intervals.

For survival analyses, all second-line regimens were regrouped into “platinum-containing,” “non-platinum-containing,” “bendamustine-containing,” and “palliative” regimens. Grouped OS curves from the start of the second-line treatment are shown in **Figure 4** and demonstrate a 5-year OS of 26%–36% for relapsed or refractory patients deemed fit for non-palliative regimens.

We presumed treatment to be for refractory (7% of all treated patients) or relapsed disease (9% of all treated patients), as defined in the methods section above. No major difference between relapsed or refractory patients in the choice of second-line regimen could be observed.

**Figure 5:**
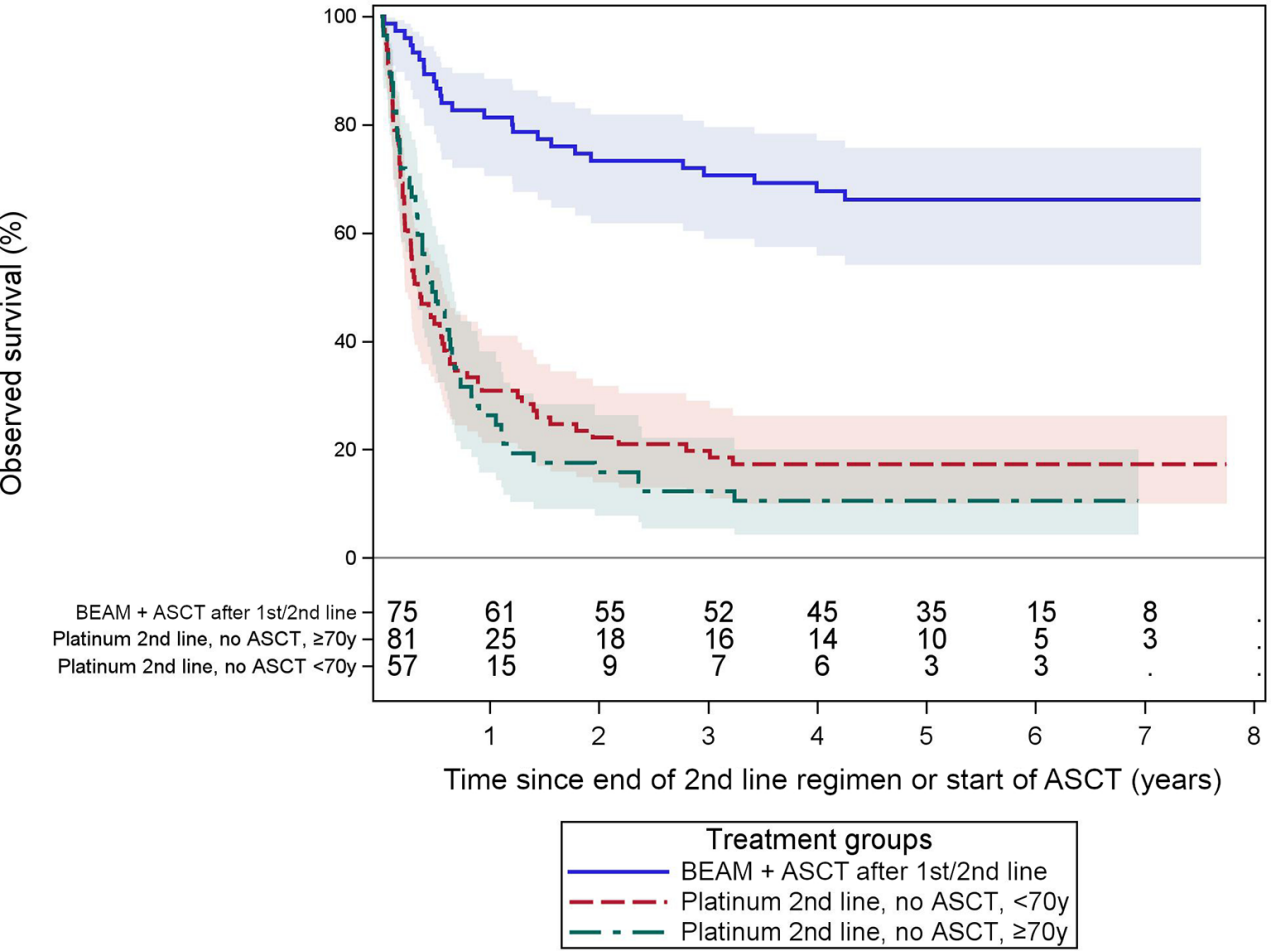
Observed survival after ASCT or platinum-containing second-line treatment without ASCT. These Kaplan–Meier curves show the observed survival from the start of ASCT or end of second-line therapy in patients either receiving an ASCT after a BEAM-like conditioning or patients receiving platinum-based salvage regimens without ASCT. The latter stratified by age (< or ≥70 years old). Observed survival in the groups without ASCT is poor compared to the ASCT group. The numbers of patients at risk are tabled below the curves. Colored areas represent the 95% confidence intervals.

**Figure 5** shows the observed survival of patients receiving a platinum-based second-line regimen without ASCT, compared to recipients of an ASCT with a BEAM-like conditioning after any preceding line. The ASCT group had a relatively good 5-year OS of 66% [54.1, 75.7]. This is in sharp contrast to those receiving salvage therapy without subsequent ASCT, with a 5-year OS of only 17% [10.0, 26.2] and 11% [4.3, 20.0] in patients aged <70 yr (n = 81) or ≥70 yr (n = 57) respectively.

**Figure 6:**
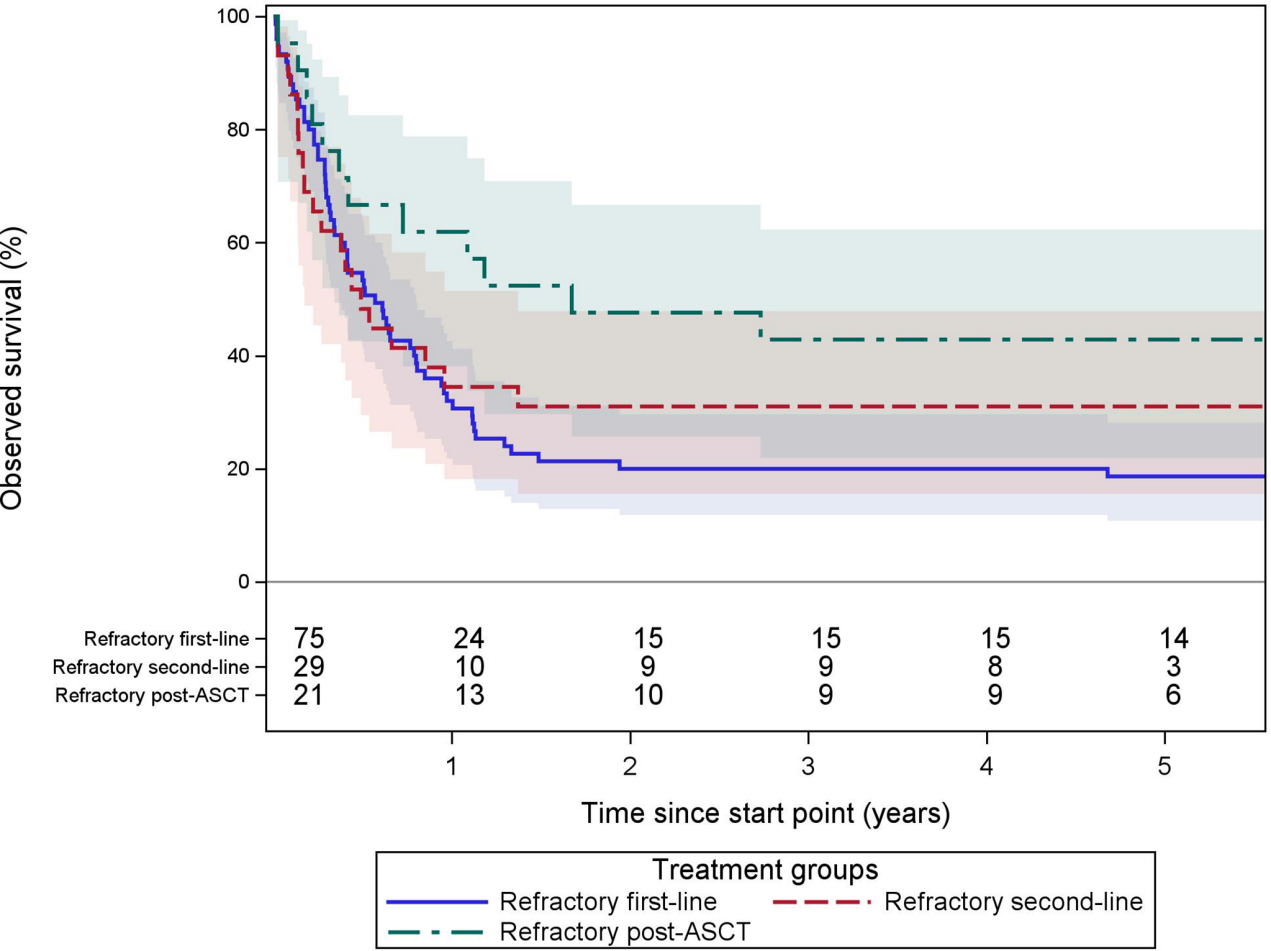
Observed survival of refractory DLBCL patients. These Kaplan–Meier curves show the observed survival from the start of second- or further-line of therapy stratified in 3 groups. In blue, patients starting any second-line regimen <12 weeks after the end of ≥4 cycles of any first line regimen (n = 75). In red, patients starting a third line regimen <12 weeks after ≥2 cycles of any second-line regimen (n = 29). In green, patients starting any therapy (radiotherapy, chemotherapy, or HSCT) <12 months after start of ASCT (ASCT within 2 years from incidence) (n = 23). The starting point for each group is different and defined as the start of the first (salvage) therapy after becoming refractory. Treatments were only considered during the 2 years of follow-up after for diagnosis. The numbers of patients at risk are tabled below the curves. Colored areas represent the 95% confidence intervals.

In an effort to approach the definition of refractory DLBCL according to the SCHOLAR-1 study (17), we analyzed 3 subgroups: first, patients starting any second-line regimen <12 weeks after the end of ≥4 cycles of any first-line regimen (n = 75); second, patients starting a third-line regimen <12 weeks after ≥2 cycles of any second-line regimen (n = 29); and third, patients starting any therapy (radiotherapy, chemotherapy, or HSCT) <12 months after the start of ASCT (only ASCT within 2 years after incidence were included) (n = 23). Overall survival is shown in **Figure 6** and **Table 3**. To be cautiously interpreted because of the selection bias due to inherent exclusion of untreated refractory patients.

**Table 3:** Observed survival of refractory DLBCL according to approximations of the SCHOLAR-1 (17) definitions.

	At 2 years		At 5 years		Median OS (years)
	Estimate	95% CI	Estimate	95% CI	
All refractory DLBCL cases	28.57	[20.5, 37.1]	26.61	[18.8, 35.1]	0.7
**Refractory per SCHOLAR-1 def.**	.		.		
Refractory at first-line	20.00	[11.9, 29.7]	18.67	[10.8, 28.2]	0.6
Refractory at second-line	31.03	[15.6, 47.9]	31.03	[15.6, 47.9]	0.5
Refractory at post-ASCT	47.62	[25.7, 66.7]	42.86	[21.9, 62.3]	1.7

The starting point for each group is different and defined as the start of the first (salvage) therapy after becoming refractory. Refractory at first-line: patients starting any second-line regimen < 12 weeks after the end of ≥ 4 cycles of any first line regimen (n = 75). Refractory at second-line: patients starting a third line regimen < 12 weeks after ≥ 2 cycles of any second-line regimen (n = 29). Refractory at post-ASCT: patients starting any therapy (radiotherapy, chemotherapy, or HSCT) < 12 months after start of ASCT (ASCT within 2 years from incidence) (n = 23). Treatments were only considered during the 2 years of follow-up after for diagnosis.

CI, confidence interval.

### 3.5 Hematopoietic Stem Cell Transplantation

#### 3.5.1 Autologous Stem Cell Transplantation

We could capture information on ASCT for 4–7 years after diagnosis for the whole cohort. In total, 82 ASCT were registered of which 67/82 within the first 2 years from diagnosis. A BEAM-like conditioning regimen was used in 91%. The ASCT was performed as part of first-line in 35/82 (43%), second-line in 44/82 (54%), and further-line in 3/82 (4%). The treatment regimen preceding the ASCT in first-line was R-CHOP(-like) in 59%, R-ACVBP in 27%, and platinum-containing in 9% of cases. In second-line, this was nearly exclusively platinum-containing (88%). The 5-year OS of 69% and 66% was similar in first- and second-line ASCT.

#### 3.5.2 Allogeneic Stem Cell Transplantation

Ten AlloSCTs for relapsed/refractory DLBCL were identified during the follow-up. They were performed after multiple lines of therapy without prior ASCT (n = 6) or at second relapse after prior ASCT (n = 4).

### 3.6 Outcome Analyses (Univariable and Multivariable Models)

The prognostic markers identified from univariable survival models with a significant HR are age category, language of the pathology report, PS, Ann Arbor stage, non-GCB COO, BCL2 overexpression, Ki-67, any considered comorbidity, prior malignancies, subsequent malignancies, center volume, and all first-line treatment categories except “other anthracycline” (**Supplementary Table 8**). Double expressions of BCL2&MYC and/or *MYC* rearrangements were associated with an inferior overall survival, but we could not include these variables in our models because of the high proportion of missing data. Having no information on Ann Arbor stage, COO, BCL2, or MYC was associated with a worse or an equivalent overall survival compared to the other subgroups of these variables.

The multivariable survival analysis (**Supplementary Table 9**) included all 1,888 patients, age category, language of the pathology report, gender, PS, cell of origin, BCL2 overexpression, Ki-67, respiratory comorbidity, cardiovascular comorbidity, diabetic comorbidity, prior malignancies, subsequent malignancies, and the different first-line treatments. After adjustment, several variables seem to be linked to overall survival with a significant type 3 test: gender (HR 1.25 for males), age (HR 1.81 to 5.95 for increasing age with the youngest age group as reference), PS (poorer prognosis of all categories compared to 0–1 category, especially for the period of time following diagnosis), cell of origin (non-GCB associated with a poorer prognosis compared to GCB, only for the period beyond 1 year after incidence), respiratory and diabetic comorbidity (HR 1.41 and 1.24), prior malignancies (HR 1.34), subsequent malignancies (HR 2.50), and first-line treatment with ≥6 cycles R-CHOP (HR 0.41) or other anthracycline-containing regimens (HR 0.72) (**Table 4**).

**Table 4:** Adjusted hazard ratios (HR) from a multivariable analysis based on Cox models including age category, gender, PS, cell of origin, respiratory comorbidity, diabetes, other malignancies, and first-line treatments.

Variable	Category	Hazard ratio	95% Confidence interval	p value
Age category	60–69 years	1.81	1.40–2.35	<0.0001
*(Ref: 20–59 years)*	70–79 years	2.62	2.05–3.34	<0.0001
	80–84 years	4.13	3.18–5.35	<0.0001
	85+ years	5.95	4.53–7.82	<0.0001
Gender	Male	1.25	1.10–1.42	0.0008
Performance status early*^a^*	>1	4.56	3.43–6.06	<0.0001
Performance status late*^a^*	>1	1.88	1.53–2.30	<0.0001
BCL2 overexpression*^b^*	Yes	1.51	1.08–2.12	0.0159
Cell of origin*^b^*	Non-GCB	1.45	1.14–1.84	0.0022
Comorbidity*^c^*	Respiratory	1.41	1.15–1.73	0.0009
	Diabetes	1.24	1.05–1.46	0.0119
Other malignancies*^d^*	Before	1.34	1.07–1.68	0.0117
	After	2.50	1.96–3.20	<0.0001
First-line treatment*^e^*	Full R-CHOP*^f^*	0.41	0.33–0.52	<0.0001
	Other anthracycline	0.72	0.55–0.94	0.0143

Only those with a significant HR are shown, results on all evaluated variables can be found in **Supplementary Table 9**.

^a^Impact on OS during time period ≤0.25 (early) versus >0.25 (late) years from incidence.

^b^Impact on OS during time period >1 year from incidence. Not significant at ≤1 year.

^c^Based on reimbursed drugs in same time period.

^d^Other malignancies before or after the diagnosis of DLBCL.

^e^Each treatment has been included in the model as time dependent variable to overcome immortal time bias.

^f^R-(mini)CHOP for ≥ 6 cycles (≥ 4 if Ann Arbor stage = I).

non-GCB, non germinal center B-cell; OS, overall survival; BCL2, B-cell lymphoma 2; DLBCL, diffuse large B-cell lymphoma; PS, performance status; R-(mini)CHOP, Rituximab, Cyclophosphamide, Doxorubicin, Vincristine, Prednisolone; Ref, reference.

## 4 Discussion

This comprehensive description of real-world first- and second-line treatments is the first of its kind for any hematological malignancy in Belgium. To our knowledge, such methodology has not been published in other registry-based studies so far, with the exception of a recent study by Huang et al. in the Taiwanese population, but limited to first-line treatments only (28).

### 4.1 Advantages and Limitations of Our Methodology

A major strength of our study is the near complete coverage of all adult DLBCL patients through the obligatory national registration at the BCR, and registration of all drugs through the national mandatory health insurance. Inclusion was done regardless of insurance status, hospital, department, or received treatment. At the BCR, nearly 90% of all Hodgkin lymphoma, diffuse large B-cell lymphoma, follicular lymphoma, and Burkitt lymphoma are recorded separately by both a pathologist and an oncological care program, suggestive of near complete coverage of all cases (4). This eliminates a potential selection bias present in single- or multicenter studies, or in registries covering only part of the population, such as the SEER-Medicare database or United Kingdom’s Clinical Practice Research Datalink (25). A similarly high coverage is also present in other registries such as the Netherlands Cancer Registry (NCR — 95%), Danish Cancer Register (LYFO — 98%) and Swedish Lymphoma Registry (SLR — 95%) (23, 24, 48).

The use of raw health insurance data eliminates the need for trained registrars to extract treatment regimens from medical records, as currently performed by most registries (23, 24, 48–50). This comes with some major advantages. First, since our algorithm is based on the raw data of the individual components instead of recoded variables, additional analysis of new components or combinations can be added without the need to recode all cases. Second, registering the individual components at individual timepoints allows to evaluate certain dose reductions, incomplete regimens, or switches between them. In essence, we capture the administered regimens instead of the intention-to-treat regimens, an important difference in a predominantly older population. Third, by combining two national databases (BCR and IMA) with obligatory registration for all patients in Belgium, we cover patients from large and small centers alike, including those diagnosed at non-hematological wards and receiving treatments outside of the original hospital.

However, our methodology does have some intrinsic limitations. First, there are limitations related to missing data. No information regarding remission status is available, neither the exact timing of relapse nor remission status at death. In Belgium, causes of death are classified according to the ICD-10 classification which does not include a lymphoma-specific code. We did not have information on all the components of established prognostic markers like the IPI. A significant proportion of other prognostic criteria like Ann Arbor stage, PS, or biomarkers from pathology reports was missing (37%–92% depending on marker). Due to the initial design of the study, no information on drugs administered more than 2 years from diagnosis was registered, leading to the underestimation of late relapses. This is illustrated by the facts that 18 out of 82 ASCT were added after selective extension of our study period from 2 to 5 years after diagnosis.

Second, limitations related to the inference of treatment regimens. We performed a deduction of the intended treatments based on IMA data and thus captured only the administered treatments and not the “intention-to-treat” regimens, in contrast to results from clinical trials. The missing information on the intent of treatment is illustrated by the different survival rates within the “incomplete” R-CHOP subgroups. Identifying refractory patients according to exact definitions used in the SCHOLAR-1 study (17) was not possible because of 2 reasons. First, we defined relapsed or refractory cases based on the initiation of another therapy, thus excluding relapsed/refractory patients unable, unfit, or refusing further treatment. This is illustrated by the decrease of relapsed and refractory cases with advancing age; we presume in part due to not starting salvage therapy in elderly unfit patients. Second, we did not have information on non-reimbursed drugs such as experimental therapies in clinical trials, the preferred option in this setting, both highlighting the underestimation of the real number of cases with our methodology. We analyzed different subgroups of patients and compared them, but it is not possible to assess the efficiency of the different types of treatments due to the retrospective nature of this research. Additionally, it is not possible to compare the efficacy of 2nd lines and 3rd lines of treatment without the information of the clinical status after the previous line (complete remission, relapsed or refractory). Reasons for altering or stopping treatments could not be identified and could be progression, intolerance, or per-protocol guidance.

Despite its inherent limitations, this real-world population-based study is the first of its kind for DLBCL in Belgium. Specifically, it assesses patients usually excluded from clinical trials [advanced age, comorbidities, and other malignancies (12%)]. It provides us with a multicentered view of all patients in Belgium with little selection bias.

### 4.2 Age Appears to Remain an Important Prognostic Factor for DLBCL Patients, and We Should Consider Changing the IPI

With an HR of 1.8 to 5.9 for increasing age (with the youngest age group as reference), it remains an important discriminating factor related to survival. Decreasing with age, 5-year OS ranged from 78% to 16%, warranting the need to compare clinical trials according to the age category of the participants. When evaluating relative survival, and thus correcting for an increase in competing risks of death due to age itself, this detrimental effect of advancing age is still observed (**Supplementary Table 2**). An interesting observation is the more pronounced drop in OS around the age of 70 instead of 60, as incorporated in the IPI. This finding has already been suggested by Advani et al. in 2010 with the introduction, and later validation of the Elderly International Prognostic Index (E-IPI) (51–54). Currently, the E-IPI is not frequently used in routine practice, and only the commonly used NCCN-IPI incorporates additional age cutoffs other than 60 years of age (6). Gang et al., from the Danish Lymphoma registry, suggested the development of the DLBCL-IPI, equally adapting the age cutoff to 70 (55). Additionally, in our cohort, overall survival worsened for each age category beyond 55 years, suggesting that the incorporation of age in risk stratifications should perhaps not be dichotomous.

### 4.3 In 12% of DLCBL Patients, a Second Primary Malignancy Is Diagnosed With a Negative Impact on Prognosis: A Group Systematically Left Out of Clinical Trials

Our data suggest an age-consistent incidence of prior malignancies, but a 5%–7% of registered malignancies within 5 years after the diagnosis of DLBCL regardless of age group. This is consistent with 5.4% secondary primary malignancies beyond 1 year after DLBCL diagnosis in 25,089 patients from the Californian Cancer Registry (56). Others have found a similar or higher incidence of 13% (before) in the Swedish population, 10.9% (after) in the US population, and 15.2% (before and after) in the Japanese population (22, 24, 57). In our cohort, the malignancies after treatment for DLBCL had the biggest impact on prognosis. Those registered were quite heterogeneous with the most prevalent being prostate, lung, colorectal, and head-and-neck cancers, and acute leukemias.

### 4.4 The Majority of Patients Were Treated With R-CHOP, and Completing It Had the Best OS

The use of R-CHOP is recommended in fit patients up to 80 years of age, R-miniCHOP in patients older than 80, and modification of the anthracycline component in frail or unfit patients (6, 18, 35). Our methodology did not allow for the discrimination of R-miniCHOP. However, Hounsome et al. recently described a similar 3-year OS for patients ≥80 yr treated with R-CHOP versus R-miniCHOP in England (58). Rituximab was included in 96% of first-line treatments, and an R-CHOP-like regimen was used in 85% of all treated patients. The latter is consistent with data in the Swedish (86%) and English populations (81%) (24, 58). The remaining first-line treatments consisted of intensified regimens like R-ACVBP or platinum-containing regimens in younger patients, in contrast to the less intensive R-CVP and rituximab monotherapy in older patients. After exclusion of all untreated patients, the 5-year OS ranged between 30% and 72% according to first-line treatments. Patients completing at least 6 cycles of R-(mini)CHOP had the best prognosis. However, immortal time bias needs to be considered for this group due to inherent exclusion of unfit patients, early treatment deaths, and primary refractory cases. A similar conclusion was found by Hamlin et al. in the US population (26). Additionally, a recent Dutch registry study showed no difference in OS between 6 and 8 R-CHOP cycles (59). The reasons for not completing ≥6 R-CHOP cycles could not be determined but may include early death, treatment-related toxicities, refractory disease, limited stage disease, and part of extended and/or non-reimbursed regimens. Overall survival of “incomplete” (R-)CHOP was worse than “full” R-CHOP, but very heterogeneous when consolidative radiotherapy was taken into account (**Figure 3**). For those patients treated with radiotherapy after incomplete R-CHOP, OS is markedly better and even similar to “full” R-CHOP. These findings further support the current evidence for the curative potential of fewer cycles of R-CHOP followed by consolidative radiotherapy in selected patients. Our findings also suggest a potential role for radiotherapy alone in selected cases. Patients without registered treatments had a very poor 5-year OS of 9%. These few long-term survivors might be explained by either complete chirurgical resection of a solitary lesion or, and most likely, unsuccessful capturing of effectively administered treatments (e.g., within clinical trials) inherent to our methodology. The (lack of) success of salvage strategies in primary refractory cases is indicated by the 5-year OS of 23%.

### 4.5 Up to 16% Receive a 2nd-Line Treatment Within 2 Years; Those Not Advancing to ASCT Have a Poor Prognosis

In our cohort, 16% of patients treated with curative intent in first-line received some form of second-line therapy. This is consistent with the 11% identified in a SEER-Medicare analysis for patients ≥66 years old (60). To no surprise, this proportion decreased with advancing age. The majority of second-line regimens contained rituximab, platinum derivatives, and cytarabine. Second-line treatment was presumed to be for relapsed disease in 9% and for primary refractory disease in 7%. The reported incidence of relapsed/refractory DLBCL patients ranges between 17% and 30%, with up to 10% after EFS24 (15, 18, 38, 61, 62). This discrepancy with our cohort probably relies on 2 main factors. Firstly, we only capture patients that relapsed within 2 years from diagnosis due to our study design. Secondly, we only capture patients actually receiving second-line regimens, and not those who relapsed but were unfit for salvage therapy. The importance of the latter is demonstrated by a Danish registry study, with 66% of relapsed or refractory patients receiving no or palliative treatments (63). Overall survival of refractory patients at first- or second-line is poor and seems similar, with a median OS of 0.6 and 0.5 years, respectively (**Table 3**). These results are comparable with results from the SCHOLAR-1 study (17). For refractory patients <12 months post-ASCT, OS seems to decrease less rapidly to reach a higher plateau than the group of refractory DLBCL in first- of second-line (**Figure 6**). However, interpretations should be done with caution due to inherent exclusion of untreated refractory patients resulting in a selection bias. Additionally, treatments were only considered during the first 2 years from incidence, limiting the real number of cases in the post-ASCT group.

Survival from the end of second-line treatment of patients not able to proceed to ASCT after platinum-based second-line regimens is still very poor (5-year OS 11%–17%). This finding is consistent with most of the available literature in the post-rituximab era (15, 63, 64).

In our cohort, only a minority of those starting platinum-based salvage regimens proceeded to ASCT. This proportion was lower than the 46% reported in a Danish registry study or the 52% in the CORAL trial (63, 65). Unfortunately, the reasons for withholding ASCT are unknown but could include patients unfit for transplantation, patients not obtaining a remission after salvage therapy, and death before ASCT due to progression or toxicities of the salvage regimen. Therefore, immortal time bias needs to be taken into account when discussing survival of ASCT recipients. Together, these findings suggest that patients not able to proceed to ASCT at first relapse or for primary refractory disease, either due to refractoriness or due to fitness, most urgently need novel therapies.

### 4.6 ASCT Is Performed in 5% of DLBCL, Frequently in First-Line, With a Good 5-Year OS

In our cohort, ASCT was performed within 0.3–3.3 years from diagnosis in 5% of patients receiving any first-line treatment. This is higher than 1.3% and 1.8% as reported in the SEER-Medicare database and 1.6% reported in the Danish Cancer Registry (3, 63, 66). Moreover, ASCT was performed in 67/82 cases within 2 years from diagnosis and in 15/82 beyond 2 years in our series. Most guidelines consider ASCT in first-line to be experimental and only to be proposed for selected high-risk patients or slow-responders (18). In our study, 43% (n = 35/82) of all ASCTs were performed in first line, mostly after R-CHOP or R-ACVBP regimens. Unfortunately, we do not have information on the exact reasons for this allocation. ASCT was performed as part of a second-line in 54% (n = 44) of cases, nearly exclusively after platinum-containing salvage regimens. Somewhat surprisingly, the 5-year OS from ASCT did not seem to differ between first- and second-line and was relatively good at 69% and 66%, respectively. This is higher than the reported 3-year OS of 53%–56% or 5-year OS of 46% from other studies (38, 63, 65, 67). Within our follow-up period, 10 allogeneic HSCT specific for DLBCL were performed, all for relapsed/refractory disease of which 4 after prior ASCT.

### 4.7 With Advancing Age, Overall Survival Worsens, and Systemic Treatment Is More Often Omitted; However, Most Older Patients Are Successfully Treated With Anthracycline-Containing Regimens

In Belgium, 56% of patients were ≥70 years old and 28% ≥80 years old. Subsequently, treatment options are impeded by comorbidities and increased frailty. Based on the prognostic markers we examined in this cohort, disease characteristics did not seem to differ by age, except that they were more frequently not reported in the older population. Therefore, prognosis appears to be mainly determined by patient- and treatment-related factors. Overall, 15% of patients did not receive any systemic lymphoma treatment ranging between 5% and 54% in young versus older patients resulting in a dismal prognosis. These findings are similar to those reported in the SEER-Medicare database for patients aged ≥66 yr, with 20%–35% of patients receiving no systemic treatments with 50% of them being >80 years old (3, 26). Additionally, in our cohort, older patients were more frequently (0.5% in 20–59 yr versus 10% in ≥85 yr) treated with radiotherapy alone.

However, our results suggest that a substantial fraction of this older population still qualifies for standard R-(mini)CHOP treatment, and more importantly, still benefits from it.

Firstly, the majority of older patients (64% in ≥70 yr and 46% in ≥80 yr) are started on anthracycline-containing first-line treatments with potentially curative intent. A study from the Netherlands Cancer Registry described the proportion of anthracycline-containing regimens to be 46% in patients ≥75 yr and 34% in those ≥80 yr (49). A study from the Danish National Lymphoma Registry showed “standard treatment” to be initiated in 64% of patients, ranging from 83% among patients aged 75–79 yr to 32% among patient aged ≥85 yr (23). Secondly, the median number of R-(mini)CHOP cycles remains 6, with a median 21-day cycle length in this older population. Finally, older patients who complete R-CHOP still have a good 5-year OS relative to their age-matched peers. Several registry studies in the US, Danish, Swedish, Dutch, English, and Taiwanese populations have also demonstrated the increased overall survival in older patients receiving R-CHOP(like) first-line therapies (23–28, 58).

Except for the ≥85-yr age category, patients treated with non-anthracycline-containing regimens demonstrated a similar overall survival compared to “incomplete” R-CHOP and other anthracycline-containing regimen. Williams et al. described a similar OS for older patients treated with R-CVP versus CHOP without rituximab in the SEER-Medicare database (25). Maguire et al. demonstrated a survival benefit for R-CVP in older patients from the Californian Cancer Registry (27). Additionally, in our cohort, treatment with radiotherapy alone, or with a limited number of cycles of R-CHOP, demonstrated curative potential for a selection of patients with limited stage disease.

Second-line therapy was started in 8% of those aged ≥80 yr who had started first-line treatment, and no one received a HSCT. Their prognosis was very dismal compared to the younger patients still fit for ASCT. These second-line regimens were still predominantly platinum-based up to the age of 84. Bendamustine-containing regimens were infrequently but exclusively used in those >75 years of age but without any long-term survival.

Therefore, this specific population has two large clinical needs: firstly, the availability of less toxic, but still effective first-line regimens for those unfit for R-miniCHOP, and secondly, potent salvage options that do not necessitate consolidation with an ASCT, a treatment too toxic for the majority of DLBCL patients.

### 4.8 A Unique View on the Patterns of Care in the Belgian Adult DLBCL Population

Despite its inherent limitations, this real-world population-based study provides useful information on the pattern of care of DLBCL in Belgium. Specifically, it assesses the clinical management of patients usually excluded from clinical trials [those with advanced age (56% ≥70 yr; 28% ≥80 yr), comorbidities, and other malignancies (12%)]. It provides a multicentered view of all patients in Belgium with little selection bias. We were able to validate, retrospectively, several known prognostic markers and map the patterns of care within the Belgian population. Some known prognostic markers such as cell of origin had a less important impact on prognosis while others like other malignancies were somewhat more important than expected (22). Currently, the French multicenter real-world cohort study (REALYSA) is evaluating some of these prognostic markers in a prospective matter (68).

During our study period, most patients received the standard of care as defined by different guidelines, albeit with some differences regarding the use of radiotherapy and ASCT in first-line. The majority of DLBCL patients are aged ≥70, addressing significant challenges with regard to treatment decisions. Nonetheless, the majority still receives adequate treatment in Belgium and a significant proportion will be cured from its DLBCL.

### 4.9 Future Directions

Using our now established methodology, we will explore the patterns of care for DLBCL in more recent incidence years to determine if clinical practice has changed. Secondly, we plan to incorporate the impact of socioeconomic factors into our analyses, as they are known to have an important influence on survival (69). Finally, we will extend this methodology to other hematological malignancies, such as follicular lymphoma, and solid tumors.

## Data Availability Statement

The original contributions presented in the study are included in the article/**Supplementary Material**. Further inquiries can be directed to the corresponding author.

## Ethics Statement

Ethical review and approval were not required for the study on human participants in accordance with the local legislation and institutional requirements. Written informed consent for participation was not required for this study in accordance with the national legislation and the institutional requirements.

## Author Contributions

WD, FO, and HA contributed to the conception and design of the study. WD wrote the first draft of the manuscript. WD, MR, ES, AD, and HA performed the data extraction from the pathology reports or linked databases. MR, WD, and HA set up the in-house algorithm to infer the treatment regimens from the health insurance database. MR organized the database and performed the data curation. GM performed the statistical analyses. All authors contributed to the manuscript revision and read and approved the submitted version.

## Conflict of Interest

The authors declare that the research was conducted in the absence of any commercial or financial relationships that could be construed as a potential conflict of interest.

## Publisher’s Note

All claims expressed in this article are solely those of the authors and do not necessarily represent those of their affiliated organizations, or those of the publisher, the editors and the reviewers. Any product that may be evaluated in this article, or claim that may be made by its manufacturer, is not guaranteed or endorsed by the publisher.

## Glossary

**Table:**

ABC	activated B-cell
ALL	acute lymphoblastic leukemia
AlloSCT	allogeneic hematopoietic stem cell transplantation
ASCT	autologous stem cell transplantation
ATC	anatomical therapeutic chemical
BCL2	B-cell lymphoma 2
BCL6	B-cell lymphoma 6
BCR	Belgian Cancer Registry
BEAM	bendamustine–etoposide–AraC–methotrexate
BHS	Belgian Haematological Society
BTR	Belgian Transplant Registry
CAR-T	chimeric antigen receptor T cells
CD10	cluster of differentiation 10, neprilysin
CHOP	cyclophosphamide, hydroxydaunorubicin, oncovin, prednisolone
CNK	Code Nationa(a)l Kode
CNS	central nervous system
COO	cell of origin
DLBCL	diffuse large B-cell lymphoma
ECOG	Eastern Cooperative Oncology Group
EFS24	event-free survival at 24 months
ESR2013	age-standardized incidence rate using the European standard population of 2013
FISH	fluorescence *in situ* hybridization
GCB	germinal center B-cell
HD MTX	high-dose methotrexate
HDC	high-dose chemotherapy
HGBCL	high-grade B-cell lymphoma
HR	hazard ratio
IFRT	involved field radiotherapy
IHC	immunohistochemistry
IMA	Intermutualistic Agency
IPI	International Prognostic Index
IRF4	interferon regulatory factor 4
IT	intrathecal
IV	intravenous
KI-67	marker of proliferation Ki-67
LDH	lactate dehydrogenase
MCL	mantle cell lymphoma
MYC	MYC proto-oncogene, bHLH transcription factor
NCCN	National Comprehensive Cancer Network
NOS	not otherwise specified
OS	overall survival
PCNSL	primary central nervous system lymphoma
PMBCL	primary mediastinal B-cell lymphoma
PS	performance status
PTLD	post-transplant lymphoproliferative disease
R	rituximab
R-ACVBP	rituximab, adriamycine, cyclophosphamide, vincristine, bleomycine, prednisolone
R-CHOP	rituximab, cyclophosphamide, hydroxydaunorubicin, oncovin, prednisolone
R-CVP	rituximab, cyclophosphamide, oncovin, prednisolone
R-DHAP	rituximab, dexamethasone, high-dose Ara-C, platinum
RT	radiotherapy
SEER	Surveillance, Epidemiology and End Results
SIR	standardized incidence ratio
WHO	World Health Organization
WSR	world standard rate
YR	years
IQR	interquartile range
