# Supplementary material for: Real-World Estimation of First- and Second-Line Treatments for Diffuse Large B-Cell Lymphoma Using Health Insurance Data: A Belgian Population-Based Study
Source: Front Oncol. 2022 Feb 28;12:824704. doi: 10.3389/fonc.2022.824704 (PMC8922541; doi:10.3389/fonc.2022.824704)
Supplement: Supplementary file 1 [file DataSheet_1.pdf]

Supplementary Material

1 Supplementary figures

Supplementary Figure 1: Absolute number of registered other malignancies, grouped by time of diagnosis from incidence of DLBCL...2
Supplementary Figure 2 : Observed survival by age..3

2 Supplementary tables

Supplementary Table 1: Patient characteristics and prognostic markers by age group. 3
Supplementary Table 2: The 2- and 5-year relative survival after incidence by patient- and treatment characteristics. 6
Supplementary Table 3: Distribution of administered first-line regimens, with or without the use of rituximab...9
Supplementary Table 4: Patient characteristics of the incomplete R-CHOP subgroups compared to full R-CHOP...10
Supplementary Table 5: Patient characteristics grouped by first-line treatments. 13
Supplementary Table 6: Patient characteristics according to CNS Prophylaxis in R-CHOP(-like) treated patients, divided by mode of administration..16
Supplementary Table 7: All second-line treatments by age group..17
Supplementary Table 8: Univariable analysis based on Cox models including all evaluated variables in all patients receiving any first line treatment. 18
Supplementary Table 9: Adjusted hazard ratios (HR) from a multivariable analysis based on Cox models including all evaluated variables in all patients receiving any first line treatment. 21

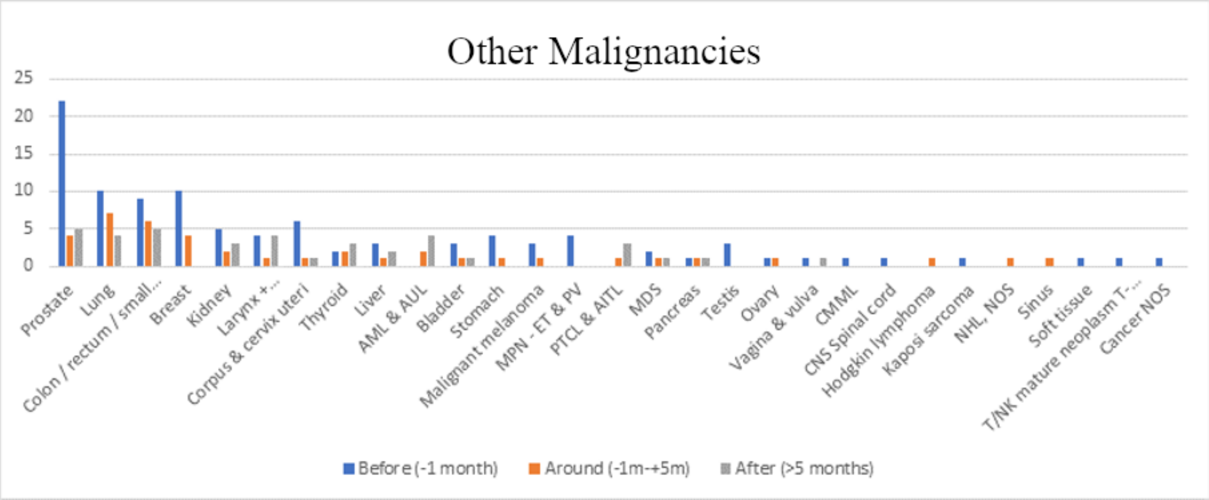

Supplementary Figure 1: Absolute number of registered other malignancies, grouped by time of diagnosis from incidence of DLBCL. Only those registered at the BCR are shown in this graph, thus excluding presumed malignancies based only on non-lymphoma treatments.

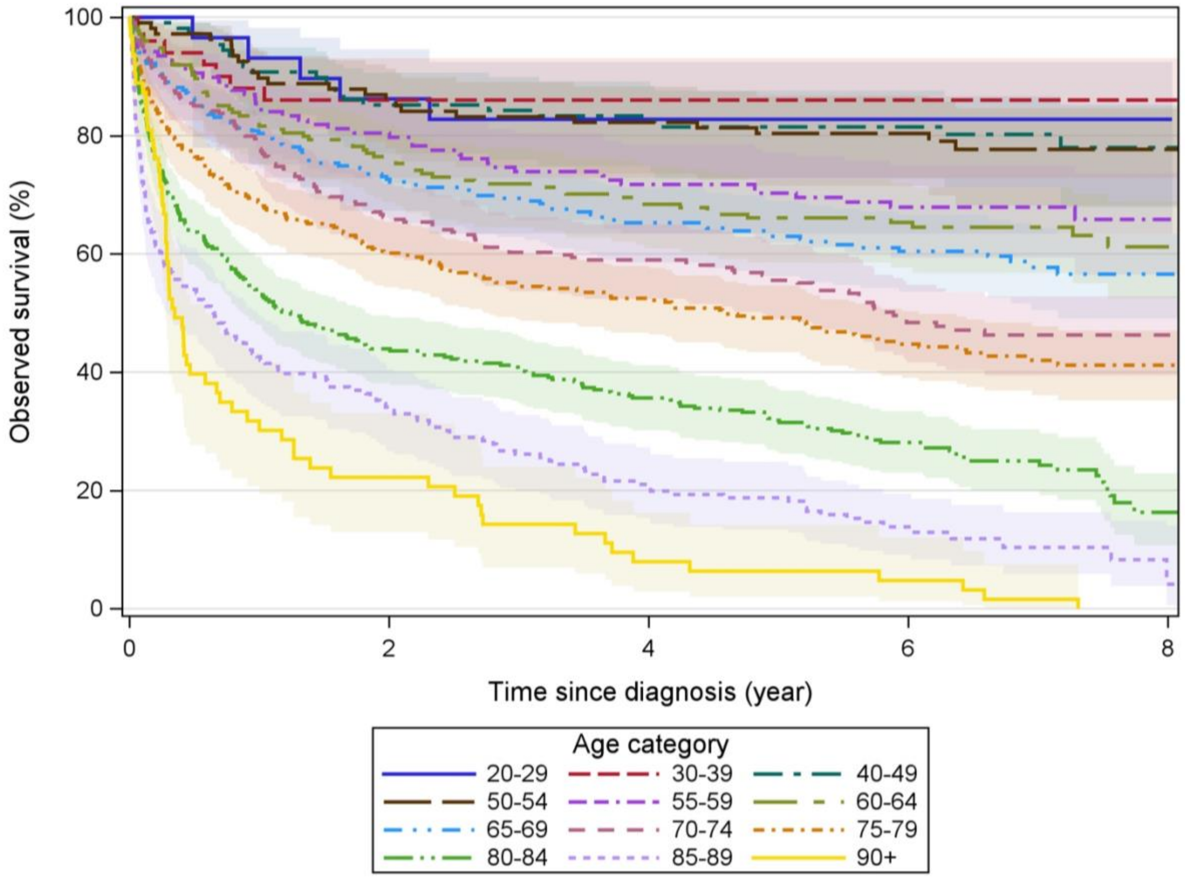

Supplementary Figure 2 : Observed survival by age. These Kaplan-Meier curves show the observed survival from time of diagnosis of all 1888 patients grouped into 12 age categories. Observed survival in the age categories below 55yr cluster together. From >55yr onward, observed survival worsens with advancing age and seems to change more markedly at the 55yr, 70yr and 80yr-mark compared to 60yr, which is the age-cutoff in the IPI risk score. Colored areas represent the 95% confidence intervals.

Supplementary Table 1: Patient characteristics and prognostic markers by age group. *PS= Performance status; GCB=Germinal Center B-cell; IHC=immunohistochemistry*

	20-59yr	60-69yr	70-79yr	80-84yr	85+yr	Overall
Overall	432	393	535	289	239	1,888
Gender						
Male	253 (59)	240 (61)	289 (54)	152 (53)	96 (40)	1,030 (55)
Female	179 (41)	153 (39)	246 (46)	137 (47)	143 (60)	858 (46)
PS						
0	82 (19)	65 (17)	63 (12)	29 (10)	10 (4)	249 (13)
1	253 (59)	227 (58)	310 (58)	147 (51)	123 (51)	1,060 (56)
2	31 (7)	32 (8)	54 (10)	39 (13)	20 (8)	176 (9)
3	12 (3)	13 (3)	20 (4)	16 (6)	24 (10)	85 (5)
4	7 (2)	6 (2)	10 (2)	5 (2)	7 (3)	35 (2)
Not available	47 (11)	50 (13)	78 (15)	53 (18)	55 (23)	283 (15)
Ann Arbor stage						
1	63 (15)	40 (10)	52 (9.72)	30 (10)	31 (13)	216 (11)
2	66 (15)	54 (14)	69 (13)	37 (13)	26 (11)	252 (13)
3	40 (9)	59 (15)	70 (13)	30 (10)	21 (9)	220 (12)
4	148 (34)	130 (33)	168 (31)	72 (25)	47 (20)	565 (30)
Not available	115 (27)	110 (28)	176 (33)	120 (42)	114 (48)	635 (34)
Cell of origin						
GCB	152 (35)	128 (33)	163 (30)	88 (30)	74 (31)	605 (32)
Not available	151 (35)	139 (35)	198 (37)	108 (37)	100 (42)	696 (37)
non-GCB	129 (30)	126 (32)	174 (33)	93 (32)	65 (27)	587 (31)
Ki-67 expression on IHC						
<50%	15 (3)	6 (2)	16 (3)	9 (3)	7 (3)	53 (3)
≥50%	256 (59)	228 (58)	294 (55)	143 (49)	123 (51)	1,044 (55)
Not available	161 (37)	159 (40)	225 (42)	137 (47)	109 (46)	791 (42)
BCL2 overexpression on IHC						
No	80 (19)	42 (11)	64 (12)	38 (13)	29 (12)	253 (13)
Not available	150 (35)	138 (35)	206 (39)	118 (41)	98 (41)	710 (38)

	20-59yr	60-69yr	70-79yr	80-84yr	85+yr	Overall
Yes	202 (47)	213 (54)	265 (50)	133 (46)	112 (47)	925 (49)
First line treatment						
Full R-CHOP	210 (49)	238 (61)	261 (49)	90 (31)	27 (11)	826 (44)
Other anthracycline	115 (27)	45 (11)	68 (13)	30 (10)	13 (5)	271 (14)
Non-anthracycline	8 (2)	16 (4)	39 (7)	46 (16)	53 (22)	162 (9)
No chemotherapy/radiotherapy	19 (4)	21 (5)	51 (10)	49 (17)	105 (44)	245 (13)
Incomplete R-CHOP	78 (18)	70 (18)	110 (21)	62 (21)	17 (7)	337 (18)
Only radiotherapy	2 (0)	3 (1)	6 (1)	12 (4)	24 (10)	47 (2)
Language of the pathology report						
French	180 (42)	126 (32)	183 (34)	91 (31)	79 (33)	659 (35)
Dutch	252 (58)	267 (68)	352 (66)	198 (69)	160 (67)	1,229 (65)
Respiratorical comorbidity						
No	402 (93)	356 (91)	487 (91)	265 (92)	210 (88)	1,720 (91)
Yes	30 (7)	37 (9)	48 (9)	24 (8)	29 (12)	168 (9)
Diabetical comorbidity						
No	407 (94)	331 (84)	435 (81)	233 (81)	207 (87)	1,613 (85)
Yes	25 (6)	62 (16)	100 (19)	56 (19)	32 (13)	275 (15)
Cardiological comorbidity						
No	336 (78)	191 (49)	165 (31)	65 (22)	34 (14)	791 (42)
Yes	96 (22)	202 (51)	370 (69)	224 (78)	205 (86)	1,097 (58)
Prior malignancy						
No	425 (98)	365 (93)	488 (91)	263 (91)	229 (96)	1,770 (94)
Yes	7 (2)	28 (7)	47 (9)	26 (9)	10 (4)	118 (6)
Subsequent malignancy						
No	409 (95)	367 (93)	499 (93)	274 (95)	229 (96)	1,778 (94)
Yes	23 (5)	26 (7)	36 (7)	15 (5)	10 (4)	110 (6)

Supplementary Table 2: The 2- and 5-year relative survival after incidence by patient- and treatment characteristics. Relative survival is calculated as the ratio of the observed survival in a group of patients to the expected survival in a comparable group of individuals from the general population matched on age, sex, region and calendar period. *PS=Performance status; GCB=Germinal Center B-cell; IHC=immunohistochemistry, CI=Confidence interval*

		2-year relative survival after incidence		5-year relative survival after incidence	
Characteristic	N	Estimate	95% CI	Estimate	95% CI
Overall	1888	66.80	[64.4, 69.1]	62.62	[59.9, 65.3]
Gender	.	.		.	
Male	1030	66.67	[63.3, 69.8]	62.30	[58.5, 66.0]
Female	858	66.92	[63.3, 70.3]	62.98	[58.9, 66.9]
PS	.	.		.	
0	249	84.55	[78.8, 89.2]	82.26	[75.3, 88.2]
1	1060	74.63	[71.5, 77.5]	70.08	[66.4, 73.6]
2	176	49.73	[41.5, 57.6]	41.21	[32.9, 49.7]
3	85	26.65	[17.5, 36.9]	26.66	[17.0, 37.7]
4	35	9.50	[3.1, 20.5]	9.16	[2.6, 21.6]
Not available	283	47.67	[41.1, 54.0]	44.46	[37.6, 51.3]
Ann Arbor stage	.	.		.	
1	216	89.48	[83.2, 94.3]	88.00	[79.9, 94.9]
2	252	80.97	[74.7, 86.2]	76.93	[69.5, 83.6]
3	220	71.80	[64.6, 78.1]	67.50	[59.3, 75.1]
4	565	60.62	[56.1, 64.8]	54.97	[50.1, 59.7]
Not available	635	56.57	[52.1, 60.8]	52.90	[48.1, 57.7]
Cell of origin	.	.		.	
GCB	605	72.77	[68.6, 76.6]	71.79	[66.9, 76.4]
Not available	696	61.74	[57.6, 65.6]	57.33	[52.8, 61.7]
non-GCB	587	66.54	[62.1, 70.7]	59.35	[54.3, 64.2]
Ki-67 expression on IHC	.	.		.	
<50%	53	71.35	[55.5, 83.4]	71.02	[53.4, 85.5]
≥50%	1044	68.93	[65.7, 72.0]	65.15	[61.4, 68.7]

		2-year relative survival after incidence		5-year relative survival after incidence	
Characteristic	N	Estimate	95% CI	Estimate	95% CI
Not available	791	63.63	[59.8, 67.3]	58.68	[54.4, 62.9]
BCL2 IHC overexpression on IHC	.	.		.	
No	253	77.33	[70.8, 82.9]	75.20	[67.8, 81.8]
Not available	710	62.30	[58.2, 66.2]	57.78	[53.3, 62.2]
Yes	925	67.34	[63.9, 70.6]	62.87	[58.9, 66.7]
Language of the pathology report	.	.		.	
French	659	65.83	[61.6, 69.8]	61.53	[56.8, 66.1]
Dutch	1229	67.31	[64.3, 70.2]	63.20	[59.8, 66.5]
Respiratorical comorbidity	.	.		.	
No	1720	68.10	[65.6, 70.5]	64.33	[61.4, 67.1]
Yes	168	53.17	[44.6, 61.2]	44.95	[36.1, 53.8]
Diabetical comorbidity	.	.		.	
No	1613	68.21	[65.6, 70.7]	64.90	[61.9, 67.8]
Yes	275	58.54	[51.9, 64.8]	49.22	[42.0, 56.4]
Cardiological comorbidity	.	.		.	
No	791	75.52	[72.1, 78.6]	73.39	[69.6, 76.9]
Yes	1097	60.12	[56.8, 63.4]	54.31	[50.5, 58.1]
Prior malignancy	.	.		.	
No	1770	67.46	[65.0, 69.9]	63.87	[61.0, 66.6]
Yes	118	56.82	[46.5, 66.2]	43.43	[32.8, 54.2]
Subsequent malignancy	.	.		.	
No	1778	65.97	[63.5, 68.4]	63.05	[60.2, 65.8]
Yes	110	79.29	[69.4, 87.0]	54.69	[43.3, 65.4]
Age category	.	.		.	
20-59yr	432	84.71	[80.9, 87.9]	79.97	[75.7, 83.6]
60-69yr	393	75.68	[70.9, 79.9]	69.07	[63.7, 74.0]
70-79yr	535	66.20	[61.6, 70.5]	62.72	[57.4, 67.8]
80-84yr	289	47.58	[40.9, 54.1]	46.51	[38.5, 54.8]

		2-year relative survival after incidence		5-year relative survival after incidence	
Characteristic	N	Estimate	95% CI	Estimate	95% CI
85+yr	239	35.10	[27.9, 42.7]	32.08	[23.0, 42.7]
First-line treatment	.	.		.	
Full R-CHOP	826	89.20	[86.4, 91.6]	83.94	[80.2, 87.3]
Other anthracycline	271	69.80	[63.5, 75.4]	66.36	[59.3, 72.8]
Non anthracycline	162	49.10	[40.2, 57.7]	42.79	[32.9, 53.1]
No chemotherapy/radiotherapy	245	6.63	[4.3, 9.6]	5.00	[3.0, 7.8]
Incomplete R-CHOP	337	57.21	[51.3, 62.7]	54.94	[48.5, 61.2]
Radiotherapy only ≤ 12 weeks	47	52.63	[34.9, 69.5]	53.05	[31.0, 77.3]

Supplementary Table 3: Distribution of administered first-line regimens, with or without the use of rituximab. n(%)

First-line regimen	With rituximab	Without rituximab
**Overall**	**1,527 (100)**	**69 (100)**
CHOP	1145 (75)	17 (25)
CHOP-like |Other	82 (5)	4 (6)
CVP	72 (5)	3 (4)
ACVBP	47 (3)	12 (17)
CODOX-M/HyperCVAD	43 (3)	1 (1)
CHOEP	42 (3)	1 (1)
COEP	26 (2)	0 (0)
Non-anthracycline |Other	22 (1)	0 (0)
rituximab monotherapy	20 (1)	0 (0)
COP	18 (1)	0 (0)
R-MAD	2 (0)	4 (6)
only Platinum-containing |Other	2 (0)	2 (3)
DHAP	2 (0)	0 (0)
Palliative	1 (0)	22 (32)
2nd line-other |Other	1 (0)	0 (0)
DHAOX	1 (0)	0 (0)
ICE	1 (0)	0 (0)
Bendamustine-containing	0 (0)	1 (1)
CNS-directed |Other	0 (0)	1 (1)
Other – Experimental |Other	0 (0)	1 (1)

Supplementary Table 4: Patient characteristics of the incomplete R-CHOP subgroups compared to full R-CHOP. Incomplete R-CHOP is defined as <6 cycles (or <4 if Ann Arbor stage=1). Refractory patients were defined as starting any second-line regimen <12 weeks from the end of the last R-CHOP cycle. *PS=Performance status; GCB=Germinal Center B-cell; IHC=immunohistochemistry; RT=radiotherapy*

	incomplete R-CHOP 4-5 cycles – no RT<12 weeks from end of last R-CHOP	incomplete R-CHOP 4-5 cycles – RT <12 weeks from end of last R-CHOP	incomplete R-CHOP <4 cycles – no RT<12 weeks from end of last R-CHOP	incomplete R-CHOP <4 cycles – RT <12 weeks from end of last R-CHOP	incomplete R-CHOP + refractory	Full R-CHOP
Overall	112	30	156	39	40	826
Gender						
Male	58 (52)	17 (57)	88 (56)	24 (62)	22 (55)	466 (56)
Female	54 (48)	13 (43)	68 (44)	15 (38)	18 (45)	360 (44)
PS						
0	22 (20)	6 (20)	12 (8)	9 (23)	2 (5)	130 (16)
1	57 (51)	19 (63)	87 (56)	23 (59)	27 (68)	527 (64)
2	11 (10)	2 (7)	20 (13)	4 (10)	4 (10)	63 (8)
3	5 (4)	0 (0)	5 (3)	0 (0)	1 (3)	22 (3)
4	4 (4)	0 (0)	5 (3)	0 (0)	1 (3)	4 (0)
Not available	13 (12)	3 (10)	27 (17)	3 (8)	5 (13)	80 (10)
Ann Arbor stage						
1	0 (0)	0 (0)	8 (5)	22 (56)	1 (3)	106 (13)
2	18 (16)	9 (30)	12 (8)	4 (10)	4 (10)	135 (16)
3	19 (17)	0 (0)	17 (11)	1 (3)	5 (13)	112 (14)
4	45 (40)	7 (23)	56 (36)	2 (5)	23 (58)	259 (31)
Not available	30 (27)	14 (47)	63 (40)	10 (26)	7 (18)	214 (26)
Cell of origin						
GCB	40 (36)	9 (30)	47 (30)	18 (46)	14 (35)	273 (33)
Not available	37 (33)	10 (33)	51 (33)	13 (33)	13 (33)	286 (35)
non-GCB	35 (31)	11 (37)	58 (37)	8 (21)	13 (33)	267 (32)

	incomplete R-CHOP 4-5 cycles – no RT<12 weeks from end of last R-CHOP	incomplete R-CHOP 4-5 cycles – RT <12 weeks from end of last R-CHOP	incomplete R-CHOP <4 cycles – no RT<12 weeks from end of last R-CHOP	incomplete R-CHOP <4 cycles – RT <12 weeks from end of last R-CHOP	incomplete R-CHOP + refractory	Full R-CHOP
Ki67						
<50	3 (3)	0 (0)	5 (3)	1 (3)	2 (5)	25 (3)
>=50	56 (50)	20 (67)	98 (63)	19 (49)	25 (63)	455 (55)
Not available	53 (47)	10 (33)	53 (34)	19 (49)	13 (33)	346 (42)
BCL2 IHC overexpression						
No	15 (13)	1 (3)	15 (10)	4 (10)	3 (8)	112 (14)
Not available	49 (44)	11 (37)	44 (28)	21 (54)	11 (28)	294 (36)
Yes	48 (43)	18 (60)	97 (62)	14 (36)	26 (65)	420 (51)
Language of the pathology report						
French	43 (38)	7 (23)	61 (39)	9 (23)	16 (40)	265 (32)
Dutch	69 (62)	23 (77)	95 (61)	30 (77)	24 (60)	561 (68)
Respiratorical comorbidity						
No	100 (89)	28 (93)	144 (92)	34 (87)	38 (95)	772 (93)
Yes	12 (11)	2 (7)	12 (8)	5 (13)	2 (5)	54 (7)
Diabetical comorbidity						
No	94 (84)	25 (83)	130 (83)	32 (82)	36 (90)	718 (87)
Yes	18 (16)	5 (17)	26 (17)	7 (18)	4 (10)	108 (13)
Cardiological comorbidity						
No	41 (37)	14 (47)	71 (46)	13 (33)	26 (65)	400 (48)
Yes	71 (63)	16 (53)	85 (54)	26 (67)	14 (35)	426 (52)
Prior malignancy						
No	107 (96)	27 (90)	145 (93)	34 (87)	36 (90)	783 (95)
Yes	5 (4)	3 (10)	11 (7)	5 (13)	4 (10)	43 (5)

	incomplete R-CHOP 4-5 cycles – no RT<12 weeks from end of last R-CHOP	incomplete R-CHOP 4-5 cycles – RT <12 weeks from end of last R-CHOP	incomplete R-CHOP <4 cycles – no RT<12 weeks from end of last R-CHOP	incomplete R-CHOP <4 cycles – RT <12 weeks from end of last R-CHOP	incomplete R-CHOP + refractory	Full R-CHOP
Subsequent malignancy						
No	106 (95)	29 (97)	151 (97)	36 (92)	38 (95)	765 (93)
Yes	6 (5)	1 (3)	5 (3)	3 (8)	2 (5)	61 (7)
Age category						
20-59yr	28 (25)	7 (23)	36 (23)	7 (18)	15 (38)	210 (25)
60-69yr	23 (21)	7 (23)	36 (23)	4 (10)	14 (35)	238 (29)
70-79yr	36 (32)	10 (33)	45 (29)	19 (49)	8 (20)	261 (32)
80-84yr	18 (16)	5 (17)	32 (21)	7 (18)	3 (8)	90 (11)
85+yr	7 (6)	1 (3)	7 (4)	2 (5)	0 (0)	27 (3)

Supplementary Table 5: Patient characteristics grouped by first-line treatments.
PS=Performance status; GCB=Germinal Center B-cell; IHC=immunohistochemistry.

	Full R-CHOP	Other anthracycline	Non-anthracycline	Incomplete R-CHOP	Only RT	No chemo radiotherapy	Overall
Overall	826	271	162	337	47	245	1,888
Gender							
Male	466 (56)	156 (58)	74 (46)	187 (55)	23 (49)	124 (51)	1,030 (55)
Female	360 (44)	115 (42)	88 (54)	150 (45)	24 (51)	121 (49)	858 (45)
PS							
0	130 (16)	40 (15)	14 (9)	49 (15)	6 (13)	10 (4)	249 (13)
1	527 (64)	158 (58)	87 (54)	186 (55)	29 (62)	73 (30)	1,060 (56)
2	63 (8)	27 (10)	16 (10)	37 (11)	3 (6)	30 (12)	176 (9)
3	22 (3)	13 (5)	13 (8)	10 (3)	2 (4)	25 (10)	85 (5)
4	4 (0)	4 (1)	3 (2)	9 (3)	0 (0)	15 (6)	35 (2)
Not available	80 (10)	29 (11)	29 (18)	46 (14)	7 (15)	92 (38)	283 (15)
Ann Arbor stage							
1	106 (13)	31 (11)	13 (8)	30 (9)	20 (43)	16 (7)	216 (11)
2	135 (16)	33 (12)	28 (17)	43 (13)	5 (11)	8 (3)	252 (13)
3	112 (14)	38 (14)	22 (14)	37 (11)	0 (0)	11 (4)	220 (12)
4	259 (31)	95 (35)	48 (30)	110 (33)	5 (11)	48 (20)	565 (30)
Not available	214 (26)	74 (27)	51 (31)	117 (35)	17 (36)	162 (66)	635 (34)
Cell of origin							
GCB	273 (33)	96 (35)	49 (30)	114 (34)	13 (28)	60 (24)	605 (32)
NA	286 (35)	91 (34)	64 (39.51)	111 (33)	24 (51)	120 (49)	696 (37)
non-GCB	267 (32)	84 (31)	49 (30)	112 (33)	10 (21)	65 (27)	587 (31)
Ki-67 expression on IHC							
<50%	25 (3)	7 (3)	6 (4)	9 (3)	1 (2)	5 (2)	53 (3)
≥50%	455 (55)	163 (60)	83 (51)	193 (57)	27 (57)	123 (50)	1,044 (55)
Not available	346 (42)	101 (37)	73 (45)	135 (40)	19 (40)	117 (48)	791 (42)

	Full R-CHOP	Other anthracycline	Non-anthracycline	Incomplete R-CHOP	Only RT	No chemo radiotherapy	Overall
BCL2 overexpression on IHC							
No	112 (14)	44 (16)	23 (14)	35 (10)	4 (9)	35 (14)	253 (13)
Not available	294 (36)	87 (32)	64 (39.51)	125 (37)	23 (49)	117 (48)	710 (38)
Yes	420 (51)	140 (52)	75 (46)	177 (53)	20 (43)	93 (38)	925 (49)
Language of the pathology report							
French	265 (32)	121 (45)	56 (35)	120 (36)	11 (23)	86 (35)	659 (35)
Dutch	561 (68)	150 (55)	106 (65)	217 (64)	36 (77)	159 (65)	1,229 (65)
Respiratorical comorbidity							
No	772 (93)	244 (90)	143 (88)	306 (91)	42 (89)	213 (87)	1,720 (91)
Yes	54 (7)	27 (10)	19 (12)	31 (9)	5 (11)	32 (13)	168 (9)
Diabetical comorbidity							
No	718 (87)	236 (87)	131 (81)	281 (83)	39 (83)	208 (85)	1,613 (85)
Yes	108 (13)	35 (13)	31 (19)	56 (17)	8 (17)	37 (15)	275 (15)
Cardiological comorbidity							
No	400 (48)	146 (54)	31 (19)	139 (41)	8 (17)	67 (27)	791 (42)
Yes	426 (52)	125 (46)	131 (81)	198 (59)	39 (83)	178 (73)	1,097 (58)
Prior malignancy							
No	783 (95)	252 (93)	151 (93)	313 (93)	41 (87)	230 (94)	1,770 (94)
Yes	43 (5)	19 (7)	11 (7)	24 (7)	6 (13)	15 (6)	118 (6)
Subsequent malignancy							
No	765 (93)	255 (94)	155 (96)	322 (96)	43 (91)	238 (97)	1,778 (94)
Yes	61 (7)	16 (6)	7 (4)	15 (4)	4 (9)	7 (3)	110 (6)

	Full R-CHOP	Other anthracycline	Non-anthracycline	Incomplete R-CHOP	Only RT	No chemo radiotherapy	Overall
Age category							
20-59yr	210 (25)	115 (42)	8 (5)	78 (23)	2 (4)	19 (8)	432 (23)
60-69yr	238 (29)	45 (17)	16 (10)	70 (21)	3 (6)	21 (9)	393 (21)
70-79yr	261 (32)	68 (25)	39 (24)	110 (33)	6 (13)	51 (21)	535 (28)
80-84yr	90 (11)	30 (11)	46 (28)	62 (18)	12 (26)	49 (20)	289 (15)
85+yr	27 (3)	13 (5)	53 (33)	17 (5)	24 (51)	105 (43)	239 (13)

Supplementary Table 6: Patient characteristics according to CNS Prophylaxis in R-CHOP(-like) treated patients, divided by mode of administration. *CNS=Central Nervous System; IT=intrathecal; IV=intravenous; MTX=methotrexate; PS=performance status. N(%)*

	MTX-IT (≤ 4 cycles)	HD MTX (1 or 2 cycles)	No CNS prophylaxis	Total
Overall	176 (14)	53 (4)	999 (81)	1,228
**Age**				
20-59yr	57 (19)	20 (7)	224 (74)	301 (25)
60-69yr	67 (21)	17 (5)	231 (73)	315 (26)
70-79yr	33 (8)	15 (4)	345 (88)	393 (32)
80-84yr	16 (10)	1 (1)	149 (90)	166 (14)
85yr+	3 (6)	0 (0)	50 (94)	53 (4)
**Sex**				
Males	98 (14)	36 (5)	557 (81)	691 (56)
Females	78 (15)	17 (3)	442 (82)	537 (44)
**PS**				
0-1	144 (16)	33 (4)	755 (81)	932 (76)
2-3-4	23 (15)	14 (9)	120 (76)	157 (13)
Not available	9 (7)	6 (4)	124 (89)	139 (11)
**Ann Arbor stage**				
1-2	34 (10)	10 (3)	289 (87)	333 (27)
3	19 (11)	5 (3)	143 (86)	167 (14)
4	79 (21)	26 (7)	277 (73)	382 (31)
Not available	44 (13)	12 (4)	290 (84)	346 (28)

Supplementary Table 7: All second-line treatments by age group (n (% of age group)).

	20-39yr	40-59yr	60-64yr	65-69yr	70-74yr	75-79yr	80-84yr	85-89yr
**Overall**	12	72	34	37	39	32	24	4
**Second-line regimen**								
2nd line-other|Other	0 (0)	0 (0)	0 (0)	1 (3)	3 (8)	1 (3)	1 (4)	1 (25)
BVAM	0 (0)	0 (0)	0 (0)	0 (0)	0 (0)	1 (3)	0 (0)	0 (0)
Bendamustine-containing	0 (0)	0 (0)	0 (0)	0 (0)	0 (0)	1 (3)	5 (21)	2 (50)
CHOP	0 (0)	0 (0)	0 (0)	0 (0)	0 (0)	1 (3)	1 (4)	0 (0)
CHOP-like|Other	0 (0)	0 (0)	0 (0)	0 (0)	0 (0)	1 (3)	0 (0)	0 (0)
CNS-directed|Other	0 (0)	1 (1)	0 (0)	0 (0)	0 (0)	1 (3)	0 (0)	0 (0)
CVP	0 (0)	1 (1)	0 (0)	0 (0)	0 (0)	1 (3)	0 (0)	0 (0)
DHAOX	1 (8)	5 (7)	4 (12)	6 (16)	2 (5)	2 (6)	2 (8)	0 (0)
DHAP	8 (67)	38 (53)	15 (44)	15 (41)	9 (23)	2 (6)	0 (0)	0 (0)
DHAP-like	0 (0)	1 (1)	4 (12)	0 (0)	3 (8)	4 (13)	0 (0)	0 (0)
GDP	0 (0)	0 (0)	0 (0)	0 (0)	1 (3)	0 (0)	0 (0)	0 (0)
GemOx	0 (0)	0 (0)	0 (0)	0 (0)	1 (3)	1 (3)	1 (4)	0 (0)
HD MTX	0 (0)	1 (1)	1 (3)	1 (3)	1 (3)	0 (0)	1 (4)	0 (0)
ICE	2 (17)	8 (11)	3 (9)	3 (8)	2 (5)	1 (3)	1 (4)	0 (0)
MINE	0 (0)	0 (0)	0 (0)	0 (0)	0 (0)	1 (3)	0 (0)	0 (0)
Non-Anthracycline |Other	0 (0)	0 (0)	0 (0)	1 (3)	0 (0)	0 (0)	0 (0)	0 (0)
Other - Experimental|Other	0 (0)	2 (3)	2 (6)	0 (0)	0 (0)	0 (0)	1 (4)	0 (0)
Palliative	0 (0)	1 (1)	0 (0)	4 (11)	6 (15)	5 (16)	3 (13)	0 (0)
R-MAD	0 (0)	8 (11)	2 (6)	0 (0)	2 (5)	1 (3)	0 (0)	0 (0)
only Cytarabine-containing	1 (8)	3 (4)	0 (0)	3 (8)	0 (0)	0 (0)	0 (0)	0 (0)
only Platinum-containing|Other	0 (0)	3 (4)	3 (9)	3 (8)	9 (23)	8 (25)	8 (33)	1 (25)

Supplementary Table 8: Univariable analysis based on Cox models including all evaluated variables in all patients receiving any first line treatment. *CI=Confidence Interval; PS=Performance status; GCB=Germinal Center B-cell; IHC=immunohistochemistry*

		Unadjusted hazard ratio				
		Lower limit of 95% CI	Estimate	Upper limit of 95% CI	P value	P value from type 3 test
Language of the pathology report		.	.	.	.	0.5997
	French (ref: Dutch)	0.91	1.04	1.18	0.5997	.
Age		.	.	.	.	<.0001
	Age (expressed in year)	1.05	1.06	1.07	<.0001	.
BCL2 overexpression on IHC [≤1 year after incidence]		.	.	.	.	0.0080
	Not available (ref: Yes) [≤1 year after incidence]	1.10	1.46	1.94	0.0093	.
	Yes (ref: No) [≤1 year after incidence]	0.88	1.17	1.54	0.2852	.
BCL2 overexpression on IHC [>1 year after incidence]		.	.	.	.	0.0020
	Not available (ref: Yes) [>1 year after incidence]	1.23	1.73	2.43	0.0018	.
	Yes (ref: No) [>1 year after incidence]	1.30	1.81	2.52	0.0004	.
Cell of origin [≤1 year after incidence]		.	.	.	.	0.0002
	Not available (ref: GCB) [≤1 year after incidence]	1.25	1.53	1.88	<.0001	.
	non-GCB (ref: GCB) [≤1 year after incidence]	0.99	1.23	1.54	0.0672	.
Cell of origin [>1 year after incidence]		.	.	.	.	0.0026
	Not available (ref: GCB) [>1 year after incidence]	1.00	1.26	1.60	0.0523	.
	non-GCB (ref: GCB) [>1 year after incidence]	1.19	1.51	1.91	0.0006	.

		Unadjusted hazard ratio				
		Lower limit of 95% CI	Estimate	Upper limit of 95% CI	P value	P value from type 3 test
Ki-67 expression on IHC [≤0.25 year after incidence]		.	.	.	.	0.0338
	Not available (ref: <50%) [≤0.25 year after incidence]	0.48	0.91	1.73	0.7765	.
	>=50% (ref: <50%) [≤0.25 year after incidence]	0.36	0.68	1.28	0.2303	.
Ki-67 expression on IHC [>0.25 year after incidence]		.	.	.	.	0.2168
	Not available (ref: <50%) [>0.25 year after incidence]	0.78	1.28	2.11	0.3335	.
	>=50% (ref: <50%) [>0.25 year after incidence]	0.86	1.42	2.34	0.1749	.
Gender		.	.	.	.	0.3935
	Male (ref: Female)	0.93	1.06	1.20	0.3935	.
PS [≤0.25 year after incidence]		.	.	.	.	<.0001
	2-3-4 (ref: 0-1) [≤0.25 year after incidence]	4.04	5.36	7.11	<.0001	.
	Not available (ref: 0-1) [≤0.25 year after incidence]	4.21	5.60	7.46	<.0001	.
PS [>0.25 year after incidence]		.	.	.	.	<.0001
	2-3-4 (ref: 0-1) [>0.25 year after incidence]	1.55	1.90	2.32	<.0001	.
	Not available (ref: 0-1) [>0.25 year after incidence]	1.00	1.25	1.56	0.0544	.
Ann Arbor stage [≤0.25 year after incidence]		.	.	.	.	<.0001
	3 (ref: 1-2) [≤0.25 year after incidence]	0.56	1.03	1.91	0.9259	.
	4 (ref: 1-2) [≤0.25 year after incidence]	1.60	2.41	3.63	<.0001	.
	Not available (ref: 1-2) [≤0.25 year after incidence]	2.81	4.13	6.07	<.0001	.

Unadjusted hazard ratio						
		Lower limit of 95% CI	Estimate	Upper limit of 95% CI	P value	P value from type 3 test
Ann Arbor stage [>0.25 year after incidence]		.	.	.	.	<.0001
	3 (ref: 1-2) [>0.25 year after incidence]	1.15	1.50	1.94	0.0026	.
	4 (ref: 1-2) [>0.25 year after incidence]	1.43	1.76	2.16	<.0001	.
	Not available (ref: 1-2) [>0.25 year after incidence]	1.31	1.61	1.99	<.0001	.
Diabetical comorbidity		.	.	.	.	<.0001
	Yes (ref: No)	1.32	1.55	1.81	<.0001	.
Cardiological comorbidity		.	.	.	.	<.0001
	Yes (ref: No)	1.83	2.10	2.40	<.0001	.
Respiratorical comorbidity		.	.	.	.	<.0001
	Yes (ref: No)	1.23	1.50	1.83	<.0001	.
Prior malignancy		.	.	.	.	<.0001
	Yes (ref: No)	1.29	1.61	2.02	<.0001	.
Subsequent malignancy		.	.	.	.	<.0001
	Yes (ref: No)	2.02	2.58	3.30	<.0001	.
Full R-CHOP		.	.	.	.	<.0001
	Yes (ref: No)	0.33	0.38	0.44	<.0001	.
Other anthracycline chemotherapy		.	.	.	.	0.3287
	Yes (ref: No)	0.75	0.91	1.10	0.3287	.
Non-anthracycline chemotherapy		.	.	.	.	<.0001
	Yes (ref: No)	2.12	2.56	3.08	<.0001	.
incomplete R-CHOP		.	.	.	.	<.0001
	Yes (ref: No)	1.28	1.50	1.75	<.0001	.
Only radiotherapy		.	.	.	.	<.0001
	Yes (ref: No)	1.79	2.49	3.47	<.0001	.

Supplementary Table 9: Adjusted hazard ratios (HR) from a multivariable analysis based on Cox models including all evaluated variables in all patients receiving any first line treatment. *CI=Confidence Interval; PS=Performance status; GCB=Germinal Center B-cell; IHC=immunohistochemistry*

		Adjusted hazard ratio				
		Lower limit of 95% CI	Estimate	Upper limit of 95% CI	P value	P value from type 3 test
Language of the pathology report		.	.	.	.	0.0715
	French (ref: Dutch)	0.99	1.13	1.30	0.0715	.
Age		.	.	.	.	<.0001
	60-69 yr (ref: 20-59 yr)	1.40	1.81	2.35	<.0001	.
	70-79 yr (ref: 20-59 yr)	2.05	2.62	3.33	<.0001	.
	80-84 yr (ref: 20-59 yr)	3.18	4.13	5.35	<.0001	.
	85+ yr (ref: 20-59 yr)	4.53	5.95	7.82	<.0001	.
BCL2 overexpression on IHC [≤1 year after incidence]		.	.	.	.	0.7114
	Not available (ref: No) [≤1 year after incidence]	0.82	1.10	1.48	0.5260	.
	Yes (ref: No) [≤1 year after incidence]	0.77	1.02	1.36	0.8888	.
BCL2 overexpression on IHC [>1 year after incidence]		.	.	.	.	0.0556
	Not available (ref: No) [>1 year after incidence]	1.00	1.43	2.04	0.0488	.
	Yes (ref: No) [>1 year after incidence]	1.08	1.51	2.12	0.0159	.
Cell of origin [≤1 year after incidence]		.	.	.	.	0.1008
	Not available (ref: GCB) [≤1 year after incidence]	1.02	1.27	1.58	0.0323	.
	non-GCB (ref: GCB) [≤1 year after incidence]	0.90	1.13	1.42	0.2862	.

		Adjusted hazard ratio				
		Lower limit of 95% CI	Estimate	Upper limit of 95% CI	P value	P value from type 3 test
Cell of origin [>1 year after incidence]		.	.	.	.	0.0090
	Not available (ref: GCB) [>1 year after incidence]	0.96	1.23	1.58	0.1027	.
	non-GCB (ref: GCB) [>1 year after incidence]	1.14	1.45	1.84	0.0022	.
Ki-67 expression on IHC [≤0.25 year after incidence]		.	.	.	.	0.4619
	Not available (ref: <50%) [≤0.25 year after incidence]	0.44	0.84	1.60	0.5915	.
	>=50% (ref: <50%) [≤0.25 year after incidence]	0.39	0.74	1.41	0.3575	.
Ki67 expression on IHC [>0.25 year after incidence]		.	.	.	.	0.6569
	Not available (ref: <50%) [>0.25 year after incidence]	0.72	1.20	1.98	0.4836	.
	>=50% (ref: <50%) [>0.25 year after incidence]	0.75	1.25	2.08	0.3927	.
Gender		.	.	.	.	0.0008
	Male (ref: Female)	1.10	1.25	1.42	0.0008	.
PS [≤0.25 year after incidence]		.	.	.	.	<.0001
	2-3-4 (ref: 0-1) [≤0.25 year after incidence]	3.43	4.56	6.06	<.0001	.
	NA (ref: 0-1) [≤0.25 year after incidence]	3.32	4.43	5.91	<.0001	.
PS [>0.25 year after incidence]		.	.	.	.	<.0001
	2-3-4 (ref: 0-1) [>0.25 year after incidence]	1.53	1.88	2.30	<.0001	.
	Not available (ref: 0-1) [>0.25 year after incidence]	0.82	1.03	1.30	0.7851	.
Diabetical comorbidity		.	.	.	.	0.0119

		Adjusted hazard ratio				
		Lower limit of 95% CI	Estimate	Upper limit of 95% CI	P value	P value from type 3 test
	Yes (ref: No)	1.05	1.24	1.46	0.0119	.
Cardiological comorbidity		.	.	.	.	0.4035
	Yes (ref: No)	0.92	1.07	1.25	0.4035	.
Respiratorical comorbidity		.	.	.	.	0.0009
	Yes (ref: No)	1.15	1.41	1.73	0.0009	.
Prior malignancy		.	.	.	.	0.0117
	Yes (ref: No)	1.07	1.34	1.68	0.0117	.
Subsequent malignancy		.	.	.	.	<.0001
	Yes (ref: No)	1.96	2.50	3.20	<.0001	.
Full R-CHOP		.	.	.	.	<.0001
	Yes (ref: No)	0.33	0.41	0.52	<.0001	.
Anthracycline-containing other than R-CHOP		.	.	.	.	0.0143
	Yes (ref: No)	0.55	0.72	0.94	0.0143	.
Non anthracycline		.	.	.	.	0.8138
	Yes (ref: No)	0.80	1.03	1.33	0.8138	.
Incomplete R-CHOP		.	.	.	.	0.5924
	Yes (ref: No)	0.74	0.94	1.19	0.5924	.
Only radiotherapy		.	.	.	.	0.6226
	Yes (ref: No)	0.62	0.91	1.33	0.6226	.
